# The application of histone deacetylases inhibitors in glioblastoma

**DOI:** 10.1186/s13046-020-01643-6

**Published:** 2020-07-18

**Authors:** Rui Chen, Mengxian Zhang, Yangmei Zhou, Wenjing Guo, Ming Yi, Ziyan Zhang, Yanpeng Ding, Yali Wang

**Affiliations:** 1 Department of Oncology, Tongji Hospital, Tongji Medical College, Huazhong University of Science and Technology, Wuhan, 430030 China; 2 Cancer Center, Integrated Hospital of Traditional Chinese Medicine, Southern Medical University, Guangzhou, 510000 Guangdong China; 3 Department of Oncology, Zhongnan Hospital, Wuhan University, Wuhan, 430030 China

**Keywords:** glioblastoma, epigenetic, histone acetylation, histone deacetylation, histone deacetylases inhibitors, gene expression

## Abstract

The epigenetic abnormality is generally accepted as the key to cancer initiation. Epigenetics that ensure the somatic inheritance of differentiated state is defined as a crucial factor influencing malignant phenotype without altering genotype. Histone modification is one such alteration playing an essential role in tumor formation, progression, and resistance to treatment. Notably, changes in histone acetylation have been strongly linked to gene expression, cell cycle, and carcinogenesis. The balance of two types of enzyme, histone acetyltransferases (HATs) and histone deacetylases (HDACs), determines the stage of histone acetylation and then the architecture of chromatin. Changes in chromatin structure result in transcriptional dysregulation of genes that are involved in cell-cycle progression, differentiation, apoptosis, and so on. Recently, HDAC inhibitors (HDACis) are identified as novel agents to keep this balance, leading to numerous researches on it for more effective strategies against cancers, including glioblastoma (GBM). This review elaborated influences on gene expression and tumorigenesis by acetylation and the antitumor mechanism of HDACis. Besdes, we outlined the preclinical and clinical advancement of HDACis in GBM as monotherapies and combination therapies.

## Background

Glioblastoma multiforme (GBM) is the most common and malignant intracranial primary tumor in adults, and it has the characteristics of substantial invasion and rapid progress. Although there is a standard combined treatment strategy of surgical plus radiotherapy and chemotherapy, the prognosis is still poor. Thus, the treatment of GBM meets severe challenges. In recent years, the view of epigenetic mechanisms contributing to diverse tumors has drawn people’s attention, and histone modification is among the well-understood examples of epigenetics.

Epigenetic changes refer to altering gene expression and cellular phenotype without modifying the DNA sequence itself. Structurally, two copies each of the core histone proteins (H2A, H2B, H3, and H4) whose N-terminal tails extend outward are wrapped around DNA and then package DNA into nucleosomes [1, 2]. Next, regular repeating structure nucleosomes compose chromatin that is the foundation for gene regulation [3]. This architecture changes when amino acid residues on the histone tails are modified by post-translational acetylation, methylation, and phosphorylation [4], leading to alteration of the distance between DNA and histones, which in turn changes the accessibility of transcription factors to gene promoter regions and finally the level of gene expression [5]. These modifications of histone N-terminal tails are determined by two kinds of enzymes. The one includes histone lysine methyltransferases (KMTs), histone acetyltransferases (HATs), and DNA methyltransferases (DNMTs). The other contains histone demethylases (KDMs), histone deacetylases (HDACs) and the ten-eleven translocation (TET) family of 5-methylcytosine oxidases [6]. The balance between these two determines chromatin architecture and then influences biological events, such as cell cycle, differentiation, and apoptosis in cancer cells [7].

Among these modifications, DNA hypermethylation and histone deacetylation in GBM has already been discovered for many years [8, 9]. But to date, no drugs that target histone methylation are approved by FDA or under clinical trials. However, another one, histone deacetylase protein is being exploited as therapeutic drug targets in various cancers, making it the focus of attention in GBM researches. Now histone deacetylase protein inhibitors, as the only one of the epigenetic agents, have been investigated in clinical trials in glioblastoma [10]. Thus, this review will firstly provide a brief overview of the effects of acetylation on gene expression and tumor phenotype, elaborate the antitumor mechanism of HDAC inhibitors, and then outline several of these promising HDACis that are in pre-clinical and clinical studies in GBM as monotherapies and combination therapies.

## Histone acetylation

There are two groups of proteins that undergo acetylation. The one includes five types of histone proteins(H1, H2A, H2B, H3, and H4) acting as the primary protein components of chromatin [11], which was first observed as early as the 1960s [12, 13]. And every one of histone proteins can be divided into three classes of three-dimensional structural motifs: the histone-fold regions, their diverse extensions, and the histone tails. These tails contain recognition sites of histone post-translational modifications, among which the reversible acetylation of histone tails has brought remarkable advances for the past few decades because of its significant role in gene expression and carcinogenesis [3]. The other is nonhistone proteins, including tumor suppressor protein p53 and the tubulin components of the cytoskeleton. They participate in many critical cellular pathways, including chromatin remodeling, cell cycle, splicing, nuclear transport, actin nucleation, and mitochondrial metabolism [14].

The balance between acetylation and deacetylation of histone is controlled by two groups of enzymes: histone acetyltransferase (HAT) and histone deacetylase (HDAC) [15]. HATs transfer acetyl groups to amino-terminal lysine residues in histones, resulting in an open and accessible chromatin structure. HDACs remove these groups oppositely, contributing to chromatin condensation and transcriptional repression [16, 17]. Herein we separately describe these two molecules as follows for a better understanding of histone acetylation and their relationship to gene expression.

### HAT

Histone acetyltransferases are a diverse set of enzymes that can be divided into two groups according to their suspected cellular origin and functions: B-type HATs, in the cytoplasm, likely catalyze acetylation events linked to the transport of newly synthesized histones from the cytoplasm to the nucleus for deposition onto newly replicated DNA [18–20]. A-type HATs, conversely in nuclear, likely catalyze transcription-related acetylation events [21]. Based on several highly conserved structural motifs of the catalytic domains, HATs contain three major families (Table 1): general control nonderepressible 5 (Gcn5)-related N-acetyltransferases (GNATs) that have members of Gcn5, PCAF, Elp3, Hat1, Hpa2, and Hpa2; MYST family comprised by primarily MOZ, Ybf2 (Sas3), Sas2 and Tip60; and p300/CBP family that consist of p300 and CBP [30]. Besides, there are other proteins like Taf1 as well as many nuclear receptor co-activators that do not contain true consensus HAT domains shown to possess intrinsic HAT activity [31].

**Table 1:** Major histone acetyltransferase families

HAT Families	Family members	HAT domain motifs^**a**^	Function domains^**b**^	HAT reaction mechanism^**c**^
GNAT	Gcn5	C-D-A-B	AT domains bromodomains	kinetic mechanism
	PCAF			
	Elp3			
	Hat1			
	Hpa2			
	Nut1			
MYST	MOZ	A	AT domains	ping-pong catalytic mechanism
	Ybf2 (Sas3)		plant homeo domains	
	Sas2		zinc finger domains	
	Tip60		chromodomains	
p300/CBP	p300	E-D-A-B	zinc finger region	Theorell–Chance mechanism
			(cys, ZZ and TAZ domain)	
	CBP		HAT domains	
			Bromodomains	

^**a**^**HAT domain motifs,** the relative positions of conserved sequence motifs in the three HAT families GNAT, MYST, and p300/CBP [22–24]. Motif A is the most highly conserved motif, which contains an Arg/Gln-X-X-Gly-X-Gly/Ala sequence that is important for acetyl-CoA recognition and binding [18];

^**b**^**Function domains,** the function domains for the GNAT, MYST and p300/CBP families of HATs. **AT(acetyltransferase) domains,** transfer acetyl groups from acetyl coenzyme A (acetyl-CoA) onto histone acceptors;

**Bromodomains;** an acetyl-lysine binding domain [21, 25, 26];

**Zinc finger domains and chromodomains;** protein:protein interaction domains that are often found in heterochromatin-associated proteins [27];

**Plant homeo domains,** a common structural motif found in all eukaryotic genomes in the nucleus. It is a Zn^2+^-binding domain involved in nucleosome/histone binding [28, 29];

^**c**^**HAT reaction mechanism,** see text for details.

The completely catalytic role of HAT families requires certain mechanisms. Respectively, GNAT family employs the kinetic mechanism that requires the formation of a ternary complex (enzyme • acetyl-CoA • H3 histone) before catalysis to guide acetyl transferred from acetyl-CoA to the substrate acceptor without the formation of a covalent enzyme intermediate [18, 32–35]. MYST family has been shown to possess a ping-pong catalytic mechanism [36], in which the acetyl group from acetyl-CoA is firstly transferred to the cysteine utilizing base glutamate deprotonates the active site cysteine. Then glutamate protonates the leaving cofactor and deprotonates the substrate lysine so that the cysteine can transfer the acetyl group to the lysine [22]. And p300/CBP family, as a represent of ‘orphan class’ of HAT enzymes that do not bind directly to DNA but are recruited to particular promoters through interactions with DNA-bound transcription factors [31], appears to hold a Theorell-Chance mechanism, in which the peptide substrate associates only very transiently with the enzyme with no need for a stable ternary complex, thus leaving as soon as the reaction is complete.

Because of HATs’ catalytic role in histone proteins and even nonhistone proteins, they may be important for normal cell proliferation, growth, and differentiation. Therefore loss or misregulation of these activities may lead to cancer. And several lines of evidence have indicated that HAT is tied to tumor suppression [37].

### HDAC

The discovery of the first HDAC almost accompanied by the first HAT [38]. Afterward, as more HDACs were found, existing 18 genes were subdivided into two types by their dependency on Zn^2+^ and four classes by homology to yeast, resulting in Zinc-dependent enzymes including class I, II and IV and Zinc-independent enzymes composed of class III HDACs (Table 2) [39, 40]. The class I HDAC family consist of HDAC1, 2, 3 and 8, which has similar homology to Rpd-3 yeast transcription factor and generally stay in the nucleus; The class II that shares homology with the yeast Hda1 protein and shuttles between the nucleus and the cytoplasm incorporates class IIa (HDAC4, 5, 7 and 9) and class IIb (HDAC6 and 10); The single HDAC11 belongs to class IV found in the nucleus that has mixed homology between Rpd-3 and Hda1 [41, 42]; And the last class III homologues of the yeast protein Sir2 is comprised of Sirt1-7 and requires NAD^+^ for their activities [43]. Among them, the first three-classes are recognized as ‘classical HDACs’ and common targets for therapy [44].

**Table 2:** Major histone deacetyltransferase families

Class of HDAC	members of each class	homology to yeast	location	HDACs as anticancer targets
class I	HDACS 1, 2, 3 and 8	RPD3 protein	in the nucleus	i) DNA-based process (DNA repair, replication and recombination) ; ii) cell-cycle progression (cell proliferation, differentiation, apoptosis) ; iii) migration; iiii) immunity. (See below for more details)
class IIa	HDACs 4, 5, 7 and 9	Hda1 protein	shuttle between the nucleus and the cytoplasm	
class IIb	HDACs 6 and 10			
class IV	HDAC11	mixed homology between Rpd-3 and Hda1	in the nucleus	
class III	(NAD+-dependent) SIRT1, 2, 3, 4, 5, 6 and 7	Sir2 protein	in the nucleus	

### Acetylation and gene expression

Stable and closed nucleosomes and chromosomal structures generally impede the access to DNA. But researches, over the past few decades, have revealed that covalent modifications of histone proteins and DNA, such as acetylation, methylation, phosphorylation and citrullination of the histone core, can fundamentally alter the organization and function of chromatin, thus regulating all DNA-based processes, like transcription [45]. Among these covalent modifications, histone acetylation is a major source of dynamic variation in chromatin structure in vivo. Multiple mechanisms of action are involved in the acetylation-dependent disruption of nucleosome array condensation. Two basic connections in chromatin compaction: histone-histone and histone-DNA interactions, are essential to stabilize the condensed chromatin. The octamer of nucleosome core is assembled by a histone (H3-H4)_2_ tetramer and two H2A-H2B dimers, around which 146 bp of DNA wraps [5]. This process requires histone-histone interactions and incorporates two steps: the first step leads to the formation of the H2A-H2B dimer and the (H3-H4)_2_ tetramer; the second step of assembly is between two H2A-H2B dimers and one (H3-H4)_2_ tetramer [46]. Besides, histone-DNA interactions that block hydrophobic histone or charged histone DNA interfaces exist in a side of double-helix DNA facing the core histone octamer and chaperones recognizing specific histone sites [47]. All of these interactions are necessary to assemble into higher-order chromatin structures, which constrain the regulation of DNA.

**Figure 1:**
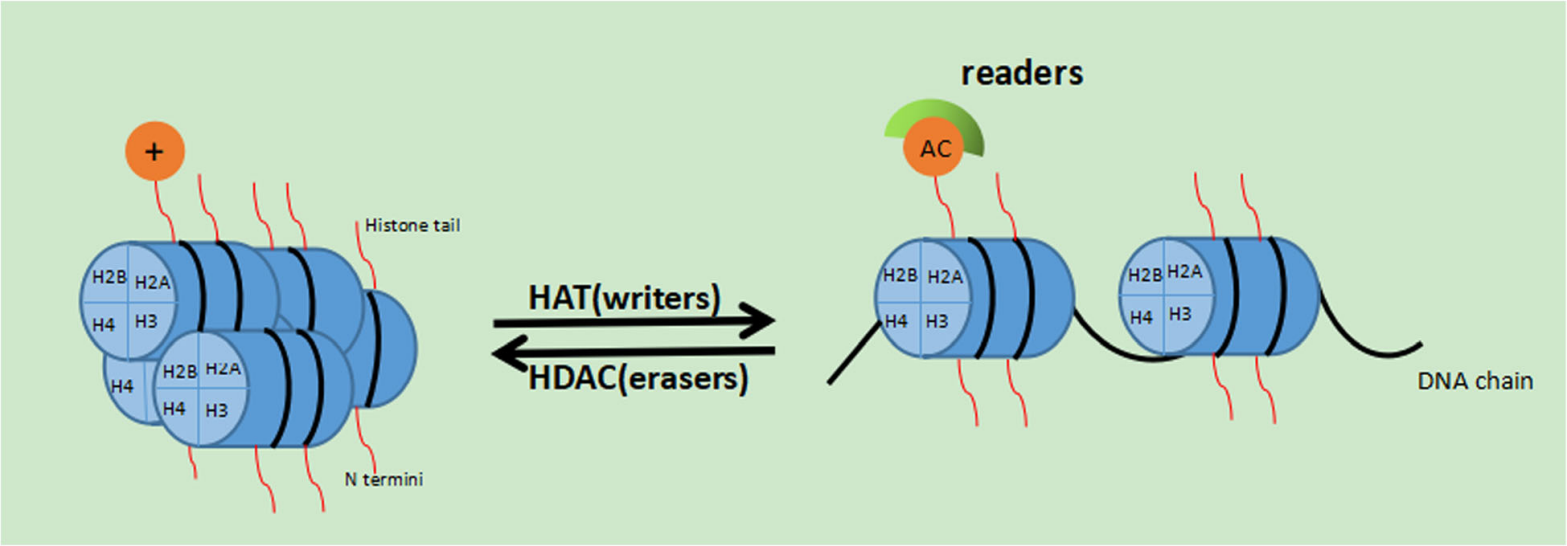
Regulation of chromatin remodeling and gene expression by acetylation. Acetylation(Ac) of the histone tills is the foundation of transcription activation. Acetyl groups on Lys residues transferred by HATs neutralizes the positively charged protruding tails, preventing the interactions with negatively charged one and thus presenting chromatin decondensation. Later this open state allows access to transcription factors and other transcription co-activators termed ‘readers’ by their contained bromodomain recognizing acetylation site on histone tails

The packaging of DNA within the tightly folded chromosomes burns a major barrier to transcription. Thus it is important for transcriptional machinery to change the stability and positioning of chromatin structures. Acetylation of the core histone N-termini, as one of the most studied and appreciated modifications, is widely considered to be correlated to the regulation of transcription. Many experiments show that histone acetylation and deacetylation mainly affects gene expression in the following ways (Figure 1).

Firstly, histone acetylation and deacetylation may function by changing the surrounding charge environment of the nucleosome, which will then strengthen or weaken the interactions between proteins related to gene expression and DNA, and in turn lead to altered chromatin architecture [48]. The histone N-terminal tail extending from the nucleosome core is generally rich in hydrophobic amino acids (e.g., lysine, arginine, serine, etc.), among which positively charged lysine residues is most likely to be acetylated by HAT. Then acetylated histone tail through the addition of an acyl group from an acyl-CoA to lysine residues with the positive charge by HATs will be neutralized, thus resulting in a decrease in binding affinity to the DNA backbone and a negatively charged neighboring nucleosome, which subsequently leads to a possibly decreased nucleosome stability, loose chromatin structure and more accessible underlying DNA [49]. On the contrary, HDACs remove the acetyl groups from histones, thereby getting back to the compacted chromatin and transcriptional repression state.

The second way to regulate gene expression by histone acetylation derives from the ‘histone code’ theory that has been recognized by most researches and incorporates ‘writers’, ‘readers’ and ‘erasers’ [4, 50]. It is just like a variety of different passwords when histone tails form a large number of special signals as a result of acetylation and deacetylation on different sites of it. Multiple HATs, as ‘writers’, transfer acetyl groups from acyl-CoA to histone N-terminal tails, producing the code identified by ‘readers’ that almost all contain the bromodomain [51]. Bromodomain, a structurally conserved module present in transcription-associated proteins (or rather more exact, histone chaperones), can recognize and bind to acetylated histones specifically and then changes the remodeling of nucleosomes, subsequently regulating transcription programs [51]. And these ‘readers’, including Spt6, the FACT (facilitates chromatin transcription) complex, anti-silencing function 1 (Asf1) and the chromatin assembly factor 1 (Caf1) complex, generally act by invading the nucleosome gradually and/or interacting with DNA or other proteins to control gene expression indirectly [25]. Once ‘readers’ bind to particular sites of acetylated lysines, the requisite transcription factors would recruit key regulatory DNA sequences (e.g., promoters and enhancers), leading to initiation of transcription. On the contrary, those enzymes that remove acetylated histone marks are termed as ‘erasers’, which prevent contacts between those transcription-associated proteins and histones or DNA [52].

Thirdly, there is one kind of protein called ATP-driven nucleosome remodelers, including the SWI/SNF complex and Chd1 or the RSC complex, expected to exert great effects on chromatin structure and mediate transcription factor binding [47]. These proteins use the energy of ATP hydrolysis to rearrange nucleosomal arrays and introduce superhelical torsion into nucleosomal DNA, which causes nucleosome sliding as well as topological and structural changes of chromatin, ultimately yielding a remodeled nucleosome with accessible DNA sites [52, 53]. Up to now, researches have dictated that these complexes, such as SWI/SNF complex, allow recruitment and DNA binding of TFIID to the TATA box, which is responsible for the initiation of transcription involving the crucial steps of sliding of nucleosome to a new position, changes in histone-DNA interactions and an increase in the association with transcription factors [54]. Additionally, another significant function involved in the transcriptional mechanism mediated by ATP-dependent complexes is that histone acetylation creates recognition sites for bromodomains present in these complexes [4]. In contrast, histone deacetylases associate with corepressor complexes to direct gene-specific transcriptional repression. Hence, the state of acetylation on core histone is critical for the recruitment of transcriptional coactivators by ATP-driven nucleosome, which forms another type of ‘histone code’ similar to chaperones binding to acetylated histone surface. Consequently, it means that chromatin structure and function differ depending on the composition of the histone variants [47].

In addition to these above mechanisms, many ways participate in and assist in transcription regulation by acetylation modification. So it is not just a sample static image of gene regulation, but rather a more dynamic and complex framework for the effects of acetylation on transcription in reality. What’s more, not only histone proteins are targeted by HATs and HDACs, but also are non-histone substrates including transcription factors like E2F,p53 and GATA1 activated by HATs [18, 55], and c-Myc, nuclear factor B (NF-B), Stat3, transcription factor IIE (TFIIE), the retinoblastoma protein, hypoxia-inducible factor 1 (HIF-1), as well as estrogen receptor and the androgen receptor complexes repressed by HDACs [10, 56–63]. The activities of these transcription factors can be a major determinant of gene activation or inactivation. Moreover, a wealth of studies uncovered the fact that other post-modifications including methylation, phosphorylation and ubiquitylation may have a tremendous impact on the functional activity of acetylation [11]. Once more than one of them acts on the same site on histones, a cross-talk will occur via multiple mechanisms either competitive or cooperative [64].

## HDACs and HDACis in cancer

Given the known function of histone acetylation in transcription, there is a strong need for a balance between histone acetyltransferase and (HAT) and histone deacetylase (HDAC). Shifts in this balance might have dramatic consequences on the cell phenotype [65, 66]. Aberrant recruitment of HDACs, which have been widely studied over the past few years, is tightly associated with malignancies and linked to cancer progression and drug resistance [67]. Accordingly, histone deacetylase inhibitors (HDACis) came into being a few decades ago, which are small moleculors to decrease the high level of HDACs and in turn increase the level of protein acetylation in the cancerous cell, restarting the expression of silenced tumor suppressor genes. Therefore, HDACis are now emerging as novel promising anticancer agents [68].

### HDACs in cancer

HDACs, which have various targets of histone and non-histone proteins, are in the focus of cancer researches due to their pleiotropic effects on genome functions including chromatin assembly, DNA repair, replication and recombination [47, 69, 70], as well as on many biological processes, such as cell proliferation, differentiation, apoptosis, and senescence [71]. Numerous evidence have demonstrated the overexpression of HDACs in diverse types of malignancies such as lung cancer [72], breast cancer [73], and hepatocellular carcinoma [74, 75]. Herein we recapitulate several excellent HDAC carcinogenic ways. For example, high level of HDAC1 has been shown to target the oncosuppressor p53 that mediates cell apoptosis [76], to prevent cells from differentiating thus maintaining an undifferentiated phenotype [77], and to enhance cell proliferation through targeting a subset of cyclin dependent kinase (CDK) inhibitors ( p21 and p27) [65]. Tip60, a DNA damage-response proteins essential for double-strand break (DSB) repair and apoptosis, might be lack of its activities when it is negatively regulated by SIRT1 and HDAC3 deacetylase activity [78–80], causing accumulation of cell damage and inactivation of the apoptotic program and finally triggering tumorigenesis [81]. The molecular chaperone heat shock protein 90 (Hsp90) is the target of HDAC6, and the involvement of HDAC6 in the Hsp-mediated regulation of VEGFR can result in an increased fibroblast cell migration [82, 83]. Also, HDAC7 overexpression might induce an increased expression of PDGF-B, which lead to angiogenesis and consequently tumor progression [84]. In addition, growing evidence supports the relation between HDACs and immune system function. Researches suggested that HDACs have a role in not only innate immunity, but also adaptive immune systems [85]. For instance, class I HDACs seem to negatively regulate innate immunity through repressing the production of an inflammatory cytokines such as COX2 [86], NF-κB [87], and IFN-β [88], and also crucially influence adaptive immunity. There is a vast array of other roles in the pathogenesis of cancer as well as other complex biological functions by HDACs. A case in point is the repression of tumour suppressor genes like p21 and the upregulation of oncogenes such as BCL2 by HDAC-mediated deacetylation [89]. Also, many studies over the years indicated that overexpression of individual HDACs correlated with poor cancer patient prognosis independent of other variables such as tumor type and disease progression. For example, elevated HDAC2 levels may be of high relevance to worse prognosis in patients with colorectal cancer [90]. Nevertheless, HDAC overexpression is not always a negative prognostic marker. Indeed, downregulated expression of HDAC10 in non-small lung carcinoma cells is reported to be related to poor prognosis in lung cancer patients [91].

### HDACis

Based on the above introduction, HDACs-mediated deacetylation is responsible for altering a large number of genes implicated in tumorigenesis. For an optimal transcription of these genes, proteins should be in an appropriate acetylated state. Hence, inhibition of histone deacetylases as a therapeutic tool in cancer emerged as a novel class of targeted drugs, which exert an antitumor effect in vitro and in vivo including the induction of the growth arrest, differentiation and apoptosis, and the inhibition of angiogenesis, DNA repair and immunomodulatory activities [92]. For instance, shreds of evidence indicate that suberic bishydroxamate (SBHA), a HDAC inhibitor, induces apoptosis by changing the balance between proapoptotic and antiapoptotic proteins in melanoma cells, which means the overexpression of proapoptotic proteins of Bcl-2 family, such as Bim, Bmf, Bax, Bak and Bik, and the repression of anti-apoptotic proteins of Bcl-2 family, such as Bcl-2, Bcl-XL, Bcl-w and Mcl-1 [93]. Also there are some other ways to induce tumour cell death, like autophagy and death receptor pathways. One of the classic HDACis, suberoylanilide hydroxamic acid (SAHA), downregulates AKT-MTOR signaling (a major suppressive cascade of autophagy) that triggers glioblastoma cell death [94]. In addition, HDACis can downregulate the expression of Nanog, a pluripotency regulator that has been shown to promote cancer progression by regulating CSCs (cancer stem cells) [95]. Table 3 provides examples grouped by pathways of alterations of those genes that play a major role in cancer (see details in Table 3).

**Table 3:** Role of HDACis in Cancer

pathway	genes/signalings	tumours affected	representative drug
**apoptosis**	mutant p53 ↓ [96, 97]	TNBC and pancreatic cancer	SAHA, NaB , VPA and TSA
	proapoptotic proteins of Bcl-2 family, such as Bim, Bmf, Bax, Bak and Bik ↑ antiapoptotic proteins of Bcl-2 family, such as Bcl-2, Bcl-XL, Bcl-w and Mcl-1 ↓ [93]	melanoma	SBHA
	XIAP ↓ [98, 99]	Mesothelioma and leukemia	LBH589 and LAQ824
	TBP2 ↑-Trx ↓-ASK1 signaling ↑ [100]	prostate cancer	SAHA
	ROS ↑ [101]	CLL	MS-275
	TRAIL-DR5 ↑ FASL-FAS (Apo-1 or CD95) ↑ TNF-TNFR-1 ↑ [102, 103]	leukaemia, breast cancer	VPA, SAHA and TSA
	human RAD23 homolog B (HR23B) ↑ [104]	U2OS cells	SAHA
	erbB2 (Her-2) ↓ [105, 106]	breast cancer	TSA and LAQ824
**cell death**	NF-κβ ↑ [107] AKT-mTOR signaling ↓ [94]	Prostate Cancer glioblastoma	SAHA SAHA
**cell arrest**	CDKN1A (encoding p21 ^WAF1/CIP1^)↑ [108–110]	CML-BC cells, colon cancer and bladder carcinoma	LAQ824, SAHA and Butyrate
	p27 ↑ [111]	leukemia and breast cancer	SAHA and TSA
	GADD45 α and GADD45 β ↑ [112]	colon carcinoma	TSA and Butyrate
	TGF-βRII ↑ - c-MYC ↓ [113]	Ewing's sarcoma (EWS) and neuroblastoma	MS-275
**angiogenesis**	HIF-1a ↓ [114] VEGF ↓ [115]	Lewis lung carcinoma HepG2 cell	FK228 TSA
**DNA repair**	Ku86 ↓ Ku70 ↓ [116]	melanoma cells	sodium butyrate (NaB)
	RAD51 ↓ BRCA1&2 ↓ [117]	human squamous carcinoma cells (SQ-20B)	TSA
**immunity**	MHC class I genes ↑ tumor antigens ↑ PD-1 ligands ↑ [118]	melanoma	LBH589, MS-275 and MGCD0103
	Treg cells ↓ [119]	renal and prostate cancer	entinostat

‘↑’ or ‘**↓**’**,** represent the up-regulated or down-regulated trend of gene expression, respectively.

## HDACis in clinic

Until now, four compounds as HDACis have already been approved by the Food and Drug Administration (FDA) for the treatment of hematological malignancy, especially cutaneous T cell lymphoma (CTCL) and peripheral T cell lymphoma (PTCL). They are vorinostat (also known as suberanilohydroxamic acid, SAHA), romidepsin, belinostat, and panobinostat. Another HDACi, Sodium Phenylbutyrate (4-PB), though not in oncology, is approved by the FDA for the treatment of urea cycle disorders, and is now being investigated for therapy in multiple types of cancer [10]. In addition, CG-745, a new clinical stage histone deacetylase (HDAC) inhibitor produced by CrystalGenomics (a biopharmaceutical company from Korea), has recently been granted as Orphan Drug Designation by FDA for pancreatic cancer (http://www.crystalgenomics.com/), which is currently in Phase II pancreatic cancer trial and the results look promising thus far. All of these encouraging results justified the introduction of more and more HDACis into clinical trials in cancer. The number of these studies that we can search on the web of Clinicaltrial.gov so far amount to 622, with more than 350 clinical trials completed or being recruiting, both as single agents and in combination with other therapeutics (https://clinicaltrials.gov/).

HDAC inhibitors that have been found to date are most the pan-HDAC inhibitors targeting multiple HDACs. They either work on Zn2+-dependent HDACs including Class I, II and IV or affect the class III HDACs that rely on NAD as a cofactor [120]. Based on their target and chemical structure, these HDACis are divided into seven categories: short chain fatty acids, benzamides, cyclic peptides, electrophilic ketones, hydro-examines, sirtuin inhibitors and miscellaneous. The common compounds of each category are shown in Table 4. The general pharmacophore essential for the activity of these HDACis includes a hydrophobic capping group for interactions with the surface of the enzyme, a linker essential for interaction with the enzymatic tunnel and connecting the cap by a small connecting unit, and the zinc binding group (ZBG) that chelates the zinc atom in the active site [147]. In these compounds, SAHA is the first and most well-known approved pan-HDACis, which was marketed under the trade name of Zolinza for the treatment of refractory cutaneous T-cell lymphoma (CTCL) in 2006. Over the years, Vorinostat has also been found to be a potent agent in the treatment of many types of cancer, such as endometrial cancer [148], lung cancer [149], gastrointestinal (GI) cancer [150] and glioblastoma [151]. And studies have shown that SAHA generally fights against cancer by upregulating the p21 (CDKN1A) cancer suppressor gene, PTEN, p27 and decreasing levels of pro-growth genes CDK2, CDK4, cyclin D1 and cyclin E [152].

**Table 4:** The classes of HDACis in clinic

target	chemical classes	compounds	source	Isotype selectivity	study phase	reference
Pan-HDAC	Hydroxamic acid	Vorinostat (SAHA)	Synthetic	class I, II and IV	FDA approval (CTCL)	[121]
		Belinostat(PXD-101)	Synthetic	class I and II	FDA approval (PTCL)	[15]
		Panobinostat (LBH-589)	Synthetic	class I, II and IV	FDA approval (PTCL and multiple myelomas)	[122]
		Trichostatin A (TSA)	Natural	class I and II	Phase I (Relapsed or Refractory hematologic malignancies )	NCT03838926
		Quisinostat (JNJ-16241199)	Synthetic	class I and II	phase II (CTCL)	NCT01486277
		WW437	Synthetic	HDAC 2 and 4	pre-clinical	[123]
	short chain fatty acids	Pivaloyloxmethyl butyrate (AN-9)	Synthetic	class I and IIa	phase II (melanoma) phase I (CLL)	NCT00087477 NCT00083473
		Sodium Butyrate (NaB)	Natural	class I and IIa	phase I (Colorectal cancer )	[124]
		Sodium Phenylbutyrate (4-PB)	Synthetic	class I and IIa	FDA approval (urea cycle disorders)	[10]
		Valproate (valproic acid)	Synthetic	class I and IIa	phase I (Brain and Central Nervous System Tumors)	[125]
	Benzamides	Entinostat (MS-275)	Synthetic	class I	phase II (Hodgkin's Lymphoma)	[126]
		Tacedinaline (CI-994)	Synthetic	class I	phase II (Myeloma)	NCT00005624
		Mocetinostat (MG-0103)	Synthetic	class I and IV	phase I (Hodgkin's Lymphoma)	[127]
	Cyclic peptides	Romidepsin (depsipeptide, FK228)	Natural	class I	FDA approval (CTCL)	[128]
	electrophilic ketones	trapoxins(TPX)	Natural	class I	NA	[129]
		a-ketoamides	Synthetic	NA	NA	[130]
		heterocyclic ketones	Synthetic	NA	NA	[131]
	miscellaneous compounds	Diallyl Trisulfide (DATS)	Natural	NA	pre-clinical (glioblastoma)	[132]
	sirtuin inhibitors	cambinol	Synthetic	SIRT1 and 2	pre-clinical	[133]
		EX-527	Synthetic	SIRT1 and 2	pre-clinical	
		sirtinol	Synthetic	SIRT1 and 2	pre-clinical	
		nicotinamide	Synthetic	class III	phase III (laryngeal cancer)	
specific HDAC	Hydroxamate Derivatives	Azelaic Bishydroxamic Acid (ABHA)	Synthetic	HDAC 3	NA	[134]
		CBHA (m-carboxycinnamic acid bis-hydroxamide)	Synthetic	HDAC 3	pre-clinical	[135]
	NA	I-7ab	Synthetic	HDAC 3	NA	[136]
		RGFP966	Synthetic	HDAC 3	pre-clinical (CTCL)	[137]
		PCI34051	Synthetic	HDAC 8	pre-clinical (T-cell lymphomas or leukemias)	[138]
		C149	Synthetic	HDAC 8	pre-clinical (T-cell lymphoma and neuroblastoma)	[139]
	Benzamides	Ricolinosta(ACY-1215)	Synthetic	HDAC 6	phase II (relapsed/refractory lymphoid malignancies )	NCT02091063
		tubacin	Synthetic	HDAC 6	pre-clinical (ALL)	[140]
	Polyketides	Depudecin	Natural	HDAC 1	NA	[71]
	sirtuin inhibitors	SEN196	Synthetic	SIRT1	NA	[141]
		COMPOUND 6J	Synthetic	SIRT2	NA	[142]
		JGB1741	Synthetic	SIRT1	NA	[143]
bromodomain	BET inhibitors	JQ1	Synthetic	BRD4	pre-clinical (CAA)	[144]
		I-BET	Synthetic	BET	pre-clinica (Breast and lung cancer)	[145]
		BY27	Synthetic	BD2	NA	[146]
hybrid molecules	chimeric	CUDC907	Synthetic	HDAC /PI3K	phase II (Thyroid Cancer)	NCT03002623
		CUDC101	Synthetic	EGFR/Her-2/HDAC 1	Phase I (head and neck, gastric, breast, liver, and non-small cell lung cancer)	NCT01171924

Though more and more HDACis have been developed, few have been used in the clinic to fight cancer, the main reasons behind which are their high toxicity and low specificity [122]. And since the toxicity is likely due to broad activity across HDAC isoforms, the development of second-generation HDAC inhibitor has been focused to improve the selectivity of HDACis, resulting in the discovery of series of specific HDACis (see details in Table 4). Until now, most of the agents developed and reported in existing articles have selectivity for HDAC3, HDAC6, HDAC8 and sirt1. For example, there are several HDAC3-selective inhibitors including RG2833 that increases the frataxin (FXN) gene silenced in Friedreich ataxia and is in a phase I clinical trial in man [153], RGFP966 that inhibits cell growth due to increased apoptosis associated with DNA damage and replication in CTCL cell lines [137], BG45 which induces the death of multiple myeloma cells concomitant with hyperacetylation and hypophosphorylation of STAT3 either singly or in combination with proteasome inhibitors [154], and I-7ab that inhibits cell viability of triple negative breast cancer (TNBC) cells and induces cell apoptosis by promoting hyperacetylation of P53 and its transcriptional activity which in turn induces the expression of p21 and consequently cause cell cycle arrest at G1 phase [155].

Besides these, there are two other special acetylation-modifying agents. The one is a small-molecule inhibitor also termed as BET (bromodomain and extra-terminal family-BRD2, BRD3, BRD4, BRDT, BD1 and BD2) inhibitor including JQ1, I-BET and more recently BY27, which disrupt the interaction between the bromodomain and acetyl-Lys. Studies show that I-BET is a type of pan BET inhibitor altering gene transcription mediated by BET proteins, and JQ1, as well as BY27, are selective BET inhibitors that competitively bind BRD4 and BD2 respectively and displaces them from chromatin [146, 156, 157]. Now many clinical trials enrolling patients with hematologic and solid tumors are ongoing, with encouraging preliminary findings [158]. The other is the molecule called a hybrid (chimeric) drug that merges two drug pharmacophores to act on different targets, of which CUDC-101 and CUDC-907 are the most representative two [95]. CUDC-101, a potent EGFR/Her-2/HDAC1 inhibitor, was developed by Cai et al. in 2010 and was found to promote tumor inhibition in various cancer xenograft models including nonsmall cell lung cancer (NSCLC), liver, breast, head and neck, colon, and pancreatic cancer [159]. Furthermore, findings strongly support that CUDC-101 has great potential to combat cancer resistance and tumor metastasis [160].

## HDACis in GBM

Virtually, almost all patients with Glioblastoma multiforme (GBM) are at the risk of recurrence, which may be ascribed to limited drug penetration of blood-brain barrier (BBB), intratumor heterogeneity, intrinsic GBM resistance, and toxicity of nonspecific agents [161]. Therefore, more targeted and effective combination strategies are urgently required for GBM treatment. Over the years, a more accurate and detailed gene expression-based molecular classification system has been built in GBM. And TCGA research network has reported three signaling pathways frequently modified in GBM, including receptor tyrosine kinase (RTK)/Ras/phosphoinositide 3-kinase (PI3K), p53, and retinoblastoma (Rb) signaling, with the mutation ratio of 88%, 87%, 78% in adults respectively. Besides, there are also variations in other genes, such as the epidermal growth factor receptor (EGFR), phosphatase and tensin homolog (PTEN) [162]. While, according to the above introduction of HDACis function, it has evoked considerable interest for the treatment of GBM. Herein, we summarize current knowledge on HDAC inhibitors’ clinical studies on GBM as monotherapies and combination therapies.

### HDACis monotherapy

#### Mechanisms of HDACis in GBM

There are several HDACis reported to be able to penetrate into BBB and play an anti-GBM role. Generally, up-regulation of HDAC proteins may be related to the occurrence and development of GBM. For instance, it has shown that the expression of HDAC9 in GBM is significantly upregulated. HDAC9 can promotes GBM proliferation and tumor formation by activating the transcription coactivator with PDZ-binding motif (TAZ), an oncogene and an essential downstream effector of the Hippo pathway [163]. So the depletion of HDAC9 can reduce the expression of TAZ, producing anti-GBM effect. Additionally, silencing of HDAC2 via its specific siRNAs can suppress the in vitro proliferation, migration, and invasion of U87 and A172 cells. Can reckon thereof, inhibitng HDAC proteins may be anti-GBM through a variety of mechanisms. According to the degree of enrichment, the mechanisms reported so far about HDACis in GBM are summarized in the following Table 5.

**Table 5:** Mechanisms of HDACis in GBM

alterations	affected part	agents
p21Waf1/Cip1, p27↑	cell cycle arrest	SAHA [151], I-BET151 [164], TSA [165], NaB [166], PB [167], FK228 [168], DATS [169], PXD-101 [170], NBM-HD-3 [171], Scriptaid [172], MS-275 [173]
DR5, TNFα, p53, Bad, Bax, Bim, chop, Puma, m-calpian↑	proapoptotic genes	SAHA [151], TSA [166], NaB [166], VPA [174], FK228 [152], DATS [169], PXD-101 [170], DWP0016 [175]
vasculogenic mimicry, VEGF, EGFR↓	angiogenesis	SAHA , MS-275, MC1568, TSA [176], NaB [177], DATS [169], LBH589 [178]
Bcl2, Bcl-XL↓	antiapoptotic genes	VPA [174], PB [167], FK228 [168], DATS [169]
EZH2, MMP-2↓	invasion	SAHA [179], VPA [174], FK228 [168], W2 [180]
p-PTEN/p-AKT, pFAK/p-STAT3↓	pathways	TSA [181], DATS [169], NBM-HD-3 [171], W2 [180]
CDK2, CDK4, CDK6, cyclins D1, cyclins D2↓	progrowth genes	SAHA [151], TSA [165]
caspase 8, caspase 9, caspase 3	apoptotic cascade activation	SAHA [182], DATS [169]
HOTAIR↓	tumor promoting lncRNA	I-BET151 [183]
Ras, c-myc↓	oncogenes	Scriptaid [172], DATS [169]
CD133, Bmi1↓	GSCs markers	SAHA [182]

‘↑’ or ‘**↓**’**,** represent the up-regulated or down-regulated trend of gene expression, respectively.

As shown in the table above, it is clear that most HDACis play an anti-GBM role by upregulating the cell cycle inhibitor p21Waf1/Cip1, thus inducing cell cycle arrest. And the second effect is on proapoptotic genes. For example, HDACis such as romidepsin and DWP0016 induce apoptosis through an increase in Bad and Bax proteins in human glioma cells in vitro [168, 175]. And exposure to belinostat in LN-229 cells leads to induction of apoptosis, associated with an increased expression of proapoptotic genes including Puma, Bim, and Chop [170]. Correspondingly, there is a decrease in anti-apoptotic genes. Researches showed that phenylbutyrate and romidepsin resulted in the reduction of anti-apoptotic proteins Bcl-xL and Bcl-2 in LN-229 cells and U251MG cells, respectively [167, 168]. Thirdly, the angiogenesis of GBM is influenced by HDACis, either by inhibiting growth factors (VEGF, EGFR) production or by blocking vascular mimicry in GBM . And more strikingly, HDACis have been proved to be efficient in targeting glioblastoma stem cells (GSCs) in the preclinical area. SAHA, TSA and valproic acid have been demonstrated to significantly trigger autophagy in GSCs , reduce proliferation rates of GSCs and stimulate differentiation in GSCs [94, 184]. To sum up, it is enough to see that the application of HDACis in GBM is promising. Here we will list some studies about single HDACis drugs investigated in GBM either in the preclinical or clinical phase as follows.

#### Single HDACis in GBM

##### Vorinostat

Vorinostat is the first HDAC inhibitor entering the trial for patients with glioma (NCT00238303). This phase II trial is studying how well vorinostat works in patients with progressive or recurrent glioblastoma multiforme who undergo surgery or do not. In general, patients received oral vorinostat (SAHA) twice daily for 14 days every three weeks. Notably, patients who undergo surgical treatment would receive oral vorinostat (SAHA) once or twice daily for a total of six doses before surgery. Finally, the trial met the primary objectives, with nine of the first 52 patients being free of progression at 6 months, and the median duration of the stable disease being 11.2 months, as well as well-tolerated toxicities. In summary, this trial shows that vorinostat has modest single-agent activity and can extend life by a few months in a subpopulation of those with recurrent glioblastoma. Nevertheless, additional testing of vorinostat in combination regimens is warranted [185].

##### Romidepsin

Based on promising preclinical data for romidepsin in glioma, North American Brain Tumor Consortium developed a phase I study to determine the maximum tolerated dose (MTD) and the pharmacokinetics of romidepsin in patients with recurrent glioma on enzyme-inducing antiepileptic drugs (EIAEDs), and a phase II study to evaluate the clinical efficacy of this drug by measuring 6-month progression-free survival and objective tumor response in patients (NCT00085540). Although the reasonably well tolerated characteristic of romidepsin in their study, the trial showed that romidepsin had no significant clinical activity as a single agent in unselected patients with recurrent GBM [186]. It is needed to find a better combination strategy for the treatment of GBM.

Currently, many HDAC inhibitors have shown considerable promise in the GBM pre-clinical phase. Still, only a few of these agents have made it into clinical trials and no one has yet to shown significant efficacy in GBM patients. Nevertheless, during studies into these drugs, researchers note that HDAC inhibitors as part of a combination therapy seem more promising in improving prognosis in this difficult to treat malignancy. So, increasing combined clinical trials in GBM about HDACis now is underway. It is worth summarizing these combination strategies.

### HDACis-involved combination therapy

There are limitations in the efficacy of single HDACis for GBM despite the tolerated toxicities that attribute to the poor pharmacokinetic properties and multiple misregulated growth and survival pathways in GBM. Thus it is rational to believe that the combinational treatment modality may represent an attractive approach to enhance the standard of care in patients with GBM. Many HDAC inhibitors are proved to act synergistically with other chemotherapy drugs, have radiosensitizing effects and enhance immunotherapies. Next, we will give an elaborate list of preclinical and clinical combination studies of HDACis for GBM in Tables 6 and 7 respectively.

**Table 6:** Combined trials of HDACis in GBM in the preclinical phase

Sensitization	HDACis	synergistic members	reference
chemotherapy	FK228	Temozolomide	[187]
	MS275	Temozolomide, etoposide, and cisplatin	[188]
	trichostatin A	lomustine	[189]
	HDAC2 inhibitor	Temozolomide	[190]
	RGFP109	Temozolomide	[191]
	Tubastatin A	Temozolomide	[192, 193]
Radiotherapy	PCI-24781	Radiation	[194]
	Tinostamustine(EDO-S101)	Radiation	[195]
	trichostatin A	Radiation	[196]
immunotherapy	J22352	PD-L1	[197]
demethylase	vorinostat or PCI-24781	LSD1	[198]
	panobinostat	DZ-Nep	[199]
BRD inhibition	panobinostat	JQ1 or OTX015	[200, 201]
RTKi	4-PB	gefitinib or vandetanib	[202]
	MS275, scriptaid, SAHA, TSA	Erlotinib	[203]
topoisomerase inhibitor	SAHA	SN38	[204]
virotherapy	trichostatin A	dl520	[205]
	Scriptaid, LBH589	Delta24-RGD	[206]
others	valproic acid (VPA)	Fluvastatin	[207]
	sodium butyrate (NaB)	quercetin	[208]
	tubastatin A	celecoxib	[209]
	panobinostat	BEZ235	[210]
	vorinostat	tranylcypromine	[211]
	SAHA	olaparib	[212]

**Table 7:** Current clinical trials of HDACis as a combination therapy in GBM

HDACis	synergistic members	conditions	phase	trial identifier	reference
Vorinostat (SAHA)	Bevacizumab	Recurrent GBM	phase II	NCT01738646	[213]
	Bevacizumab, Temozolomide	Recurrent Malignant Gliomas	phase I/II	NCT00939991	[214]
	Temozolomide	Malignant Gliomas	phase I	NCT00268385	[215]
	Radiation	Recurrent Glioma	phase I	NCT01378481	-
	Isotretinoin, Temozolomide	Recurrent GBM	phase I/II	NCT00555399	-
	Erlotinib, Temozolomide	Recurrent GBM	phase I/II	NCT01110876	-
	Temozolomide, Radiation	Newly Diagnosed GBM	phase I/II	NCT00731731	[216]
	Pembrolizumab, Temozolomide	Newly Diagnosed GBM	phase I	NCT03426891	-
	Bortezomib	Recurrent GBM	phase II	NCT00641706	[217]
	Bevacizumab, Irinotecan	Recurrent GBM	phase I	NCT00762255	[218]
Panobinostat (LBH-589)	Bevacizumab	Recurrent High Grade Glioma	phase I/II	NCT00859222	[219]
	Radiation	Recurrent Glioma	phase I	NCT01324635	-
Valproate (valproic acid, VPA)	Sorafenib Tosylate, Sildenafil Citrate	Recurrent High-Grade Glioma	phase II	NCT01817751	-
	Temozolomide, Radiation	High Grade Gliomas	phase II	NCT00302159	[220–222]
	Nivolumab, Radiation	Recurrent GBM	phase I	NCT02648633	-
Belinostat (PXD101)	Temozolomide, Radiation	GBM	phase II	NCT02137759	-

#### Preclinical

In vitro, studies have shown significant promise about HDACis synergizing with other drugs for cancer treatment [223], providing a rationale to apply these synergistic ways to GBM. Several agents have been tested in combination therapy in vitro, either as chemosensitizers or radiosensitizer, or in association with other antitumor treatments (see Table 6 for details ).

Generally, HDACis can inhibit DNA repair responses thus leading to increased DNA damage, which may partly contribute to enhanced sensitivity of tumour cells to chemotherapy and radiotherapy [224]. In GBM, studies have found that FK228 can augment temozolomide sensitivity in vivo and in vitro partially by blocking PI3K/AKT/mTOR signal pathways, triggering the cell apoptosis pathway and finally leading to cell death of glioma cell lines [187]. And histone deacetylase inhibitor RGFP109 has also been proved to be able to enhance TMZ-induced cytotoxicity in four TMZ-resistant GBM cell lines by blocking NF-κB-dependent transcription [191].

Besides, treatment with tubastatin A or ACY-1215 or CAY10603, selective HDAC6 inhibitors, were reported to abrogate temozolomide resistance by decreasing and inactivating EGFR protein, thus reducing glioblastoma clonogenicity and migration capacities, accelerating temozolomide-induced apoptosis, and finally reversing the malignant phenotype [192]. Furthermore, silencing of HDAC2 can also increase the sensitivity of GBM cells to temozolomide (TMZ), which might be due to the significant down-regulation of the multidrug resistance-associated protein 1 (MRP1) [190]. All in all, both of these research results show that HDACis can be an attractive agent to overcome chemoresistance, and combining HDACis with chemotherapy may be a promising approach to GBM.

Except for sensitization to chemotherapy, HDACis are also demonstrated to increase sensitivity to radiotherapy, modulate activities of immunity, and bolster antitumor effects of many other drugs. For example, a study indicated that TSA, a potent HDACis, could radiosensitize human glioblastoma cells [196]. And the treatment of pan-HDAC inhibitors, LBH589 (panobinostat) and suberoylanilide hydroxamic acid (SAHA, vorinostat), were shown to induce chromatin decondensation and prevent DNA DSBs repair, resulting in increased tumor cell death and radiosensitivity [225]. So clinical trials using HDACis in combination with radiotherapy should be considered useful for glioblastoma patients.

Also, there are some findings provide proof-of-principle evidence in support of a therapeutically relevant immunostimulatory activity of HDACis against GBM. For instance, a high-selective HDAC6 inhibitor, J22352, was reported to increased levels of immune-activating cytokines and the proliferation of CD8^+^ T cells by decreasing negative regulation of PD-L1, which made it possible to combine HDACis with immunotherapy to against GBM [197]. Moreover, TSA can lead GBM cells to release high-mobility group box 1 (HMGB1), an endogenous Toll-like receptor 4 (TLR4) ligand that promotes cytotoxic T-cell mediated antitumor immune responses [226].

Despite successful outcomes from these preclinical studies, only a few combination strategies have entered into clinical trials for GBM patients. Table 7 summarizes these completed or ongoing combination of clinical trials.

#### Clinical

##### Vorinostat

Vorinostat was the first HDAC inhibitor entering clinical trials in GBM, which was a phase I trial in 2005 using vorinostat together with Temozolomide (TMZ) to treat patients with malignant gliomas (NCT00268385). The primary objective of this trial is to evaluate the safety and tolerability of combining an HDAC inhibitor with TMZ in high-grade glioma (HGG). Based on the information obtained from this phase I clinical trial, a phase II trial of vorinostat with radiotherapy and concomitant TMZ later were underway (NCT00731731). This phase I/II trial demonstrated reasonable tolerability in newly diagnosed GBM. However, the primary efficacy endpoint was not met, with the OS rate at 15 months of 55.1% in the entire cohort and median OS of 16.1 months [216]. And another phase I trial of vorinostat in combination with bevacizumab and irinotecan (a topoisomerase I inhibitor) in recurrent GBM found the cumulative toxicity associated with CPT-11 and its unclear efficacy in glioblastoma, thus providing a more promising strategy for future investigation of combining vorinostat with bevacizumab alone in recurrent glioblastoma (NCT00762255) [218]. Then due to the early success of bevacizumab and subsequent approval of bevacizumab by the FDA for treatment of recurrent GBM, a phase II trial tested the efficacy of vorinostat combination with bevacizumab (NCT01738646). Ultimately, this combined treatment was tolerable, but there was no improvement in progression-free survival at 6 months [213].

##### Panobinostat

There is only two combination strategies about panobinostat in GBM approved into the clinical trial. The first is a phase II trial of panobinostat combination with bevacizumab in recurrent GBM to determine the efficacy of LBH589 by measuring 6-month progression-free survival (PFS6) (NCT00859222). It finally turned out to be well-tolerated, but it did not significantly improve PFS6 compared with bevacizumab monotherapy in the cohort [219]. The other one is a phase I trial for recurrent glioma combining panobinostat with stereotactic radiation treatment, which was terminated because of the poor accrual (NCT01324635).

##### Valproic acid

A phase II trial investigated the effects of combination treatment of VPA, temozolomide and concurrent radiotherapy for GBM patients, which shown promising results (NCT00302159). This study demonstrated improved outcomes compared to historical data and merits with the median OS of 29.6 months (range: 21–63.8 months), median progression-free survival (PFS) of 10.5 months (range: 6.8–51.2 months) and tolerated toxicities in patients with newly diagnosed GBM [222]. However, another phase I study on patients with recurrent GBM using romidepsin was terminated for the reason that pharmaceutical company (BMS) would no longer provide nivolumab for this study (NCT02648633).

## Perspectives and conclusion

Even though our progress in understanding the function of HDACs in tumour pathogenesis and the tumour response to HDACi are fruitful, there is still more hope for the exploitation of this knowledge to develop more effective clinical protocols. The clinical trials about HDACis currently achieve little for GBM treatment, and a better application strategy is urgent required. There are areas that we have not covered and might become relevant in the future. For instance, due to the heterogeneity of GBM tumors, the exist of GSCs, and the sophisticated genetic, epigenetic, and transcriptional profiling in GBM, it is difficult to identify patients who are most likely to respond to HDACis and identify specific biomarkers relative to therapeutic effects. Furthermore, the relationship between the toxicity of HDACis and their pharmacodynamic/pharmacokinetic properties is still mostly unknown, which makes it challenging to convert more viable preclinical studies into clinical trials for the further possible regimens of GBM. And so far, few research had addressed the role of HDACis in GSCs sensitivity, which should be on the agenda to clarify the true potential of HDACi in clinical treatment.

In all, only by fulling understanding the underlying molecular mechanisms can we translate these scientific findings into effective clinical practices to anti-cancer strategies. We should pursue new discoveries to advance GBM treatment.

## Data Availability

All data generated or analysed during this study are included in this published article.

## Abbreviations

TNBC: Triple-negative breast cancer
CML-BC: Human chronic myeloid leukemia blast crisis cells
XIAP: X-linked inhibitor of apoptosis
TBP2: Trx binding protein 2
ASK1: Apoptosis signal regulating kinase1;
ROS: Reactive oxygen species
CLL: Chronic lymphocytic leukemia
TRAIL: Tumour-necrosis factor-related apoptosis-inducing ligand
DR5: Death receptor 5
TNF: Tumor necrosis factor
VPA: Valproic acid
TSA: Trichostatin A
AIF: Apoptosis-inducing factor
ALL: Acute lymphoblastic leukemia
CAA: Cholangiocarcinoma
EZH2: Enhancer of Zeste 2
MMPs: metalloproteinases
HOTAIR: lncRNA HOX transcript antisense RNA
MRP1: multidrug resistance-associated protein 1
HMGB1: high-mobility group box 1
TLR4: Toll-like receptor 4

## References

[1] Richmond TJ, Davey CA (2003). The structure of DNA in the nucleosome core. Nature.

[2] Luger K (2003). Structure and dynamic behavior of nucleosomes. Curr Opin Genet Dev.

[3] Luger K, Richmond TJ (1998). The histone tails of the nucleosome. Curr Opin Genet Dev.

[4] Strahl BD, Allis CD (2000). The language of covalent histone modifications. Nature.

[5] Wolffe AP, Hayes JJ (1999). Chromatin disruption and modification. Nucleic Acids Res.

[6] Jones PA, Issa JP, Baylin S (2016). Targeting the cancer epigenome for therapy. Nat Rev Genet.

[7] Marks PA, Richon VM, Rifkind RA. Histone deacetylase inhibitors: inducers of differentiation or apoptosis of transformed cells.; 2000. pp 1210-1216. 10.1093/jnci/92.15.1210 10922406

[8] Nagarajan RP, Costello JF (2009). Epigenetic mechanisms in glioblastoma multiforme. Semin Cancer Biol.

[9] Li P, Wu M. Epigenetic Mechanisms of Glioblastoma. 2017. 29251856

[10] Lee P, Murphy B, Miller R, Menon V, Banik NL, Giglio P, Lindhorst SM, Varma AK, Vandergrift WR, Patel SJ, Das A (2015). Mechanisms and clinical significance of histone deacetylase inhibitors: epigenetic glioblastoma therapy. Anticancer Res.

[11] Kouzarides T. Chromatin modifications and their function.; 2007. pp 693-705. 10.1016/j.cell.2007.02.005 17320507

[12] JOHNS EW, PHILLIPS DM, SIMSON P, BUTLER JA (1961). The electrophoresis of histones and histone fractions on starch gel. Biochem J.

[13] PHILLIPS DM (1963). The presence of acetyl groups of histones. Biochem J.

[14] Dancy BM, Cole PA (2015). Protein lysine acetylation by p300/CBP. Chem Rev.

[15] Mottamal M, Zheng S, Huang TL, Wang G (2015). Histone deacetylase inhibitors in clinical studies as templates for new anticancer agents. Molecules.

[16] Batty N, Malouf GG, Issa JP (2009). Histone deacetylase inhibitors as anti-neoplastic agents. Cancer Lett.

[17] Parbin S, Kar S, Shilpi A, Sengupta D, Deb M, Rath SK, Patra SK (2014). Histone deacetylases: a saga of perturbed acetylation homeostasis in cancer. J Histochem Cytochem.

[18] Roth SY, Denu JM, Allis CD (2001). Histone acetyltransferases. Annu Rev Biochem.

[19] Ruiz-Carrillo A, Wangh LJ, Allfrey VG (1975). Processing of newly synthesized histone molecules. Science.

[20] Allis CD, Chicoine LG, Richman R, Schulman IG (1985). Deposition-related histone acetylation in micronuclei of conjugating Tetrahymena. Proc Natl Acad Sci U S A.

[21] Brownell JE, Allis CD (1996). Special HATs for special occasions: linking histone acetylation to chromatin assembly and gene activation. Curr Opin Genet Dev.

[22] Yan Y, Barlev NA, Haley RH, Berger SL, Marmorstein R (2000). Crystal structure of yeast Esa1 suggests a unified mechanism for catalysis and substrate binding by histone acetyltransferases. Mol Cell.

[23] Dutnall RN, Tafrov ST, Sternglanz R, Ramakrishnan V (1998). Structure of the histone acetyltransferase Hat1: a paradigm for the GCN5-related N-acetyltransferase superfamily. Cell.

[24] Neuwald AF, Landsman D (1997). GCN5-related histone N-acetyltransferases belong to a diverse superfamily that includes the yeast SPT10 protein. Trends Biochem Sci.

[25] Dhalluin C, Carlson JE, Zeng L, He C, Aggarwal AK, Zhou MM (1999). Structure and ligand of a histone acetyltransferase bromodomain. Nature.

[26] Wolffe AP, Pruss D (1996). Targeting chromatin disruption: Transcription regulators that acetylate histones. Cell.

[27] Jones DO, Cowell IG, Singh PB (2000). Mammalian chromodomain proteins: their role in genome organisation and expression. Bioessays.

[28] Bienz M. The PHD finger, a nuclear protein-interaction domain. Trends Biochem Sci 2006;31:35-40. 10.1016/j.tibs.2005.11.001 16297627

[29] Morra R, Lee BM, Shaw H, Tuma R, Mancini EJ (2012). Concerted action of the PHD, chromo and motor domains regulates the human chromatin remodelling ATPase CHD4. Febs Lett.

[30] Kelly RD, Cowley SM (2013). The physiological roles of histone deacetylase (HDAC) 1 and 2: complex co-stars with multiple leading parts. Biochem Soc Trans.

[31] Kimura A, Matsubara K, Horikoshi M (2005). A decade of histone acetylation: marking eukaryotic chromosomes with specific codes. J Biochem.

[32] Tanner KG, Trievel RC, Kuo MH, Howard RM, Berger SL, Allis CD, Marmorstein R, Denu JM (1999). Catalytic mechanism and function of invariant glutamic acid 173 from the histone acetyltransferase GCN5 transcriptional coactivator. J Biol Chem.

[33] Tanner KG, Langer MR, Kim Y, Denu JM (2000). Kinetic mechanism of the histone acetyltransferase GCN5 from yeast. J Biol Chem.

[34] Tanner KG, Langer MR, Denu JM (2000). Kinetic mechanism of human histone acetyltransferase P/CAF. Biochemistry-us.

[35] Lau OD, Courtney AD, Vassilev A, Marzilli LA, Cotter RJ, Nakatani Y, Cole PA (2000). p300/CBP-associated factor histone acetyltransferase processing of a peptide substrate. Kinetic analysis of the catalytic mechanism. J Biol Chem.

[36] McCullough CE, Marmorstein R (2016). In Vitro Activity Assays for MYST Histone Acetyltransferases and Adaptation for High-Throughput Inhibitor Screening. Methods Enzymol.

[37] Davie JR, Samuel SK, Spencer VA, Holth LT, Chadee DN, Peltier CP, Sun JM, Chen HY, Wright JA (1999). Organization of chromatin in cancer cells: role of signalling pathways. Biochem Cell Biol.

[38] Nakajima H (2007). A mammalian histone deacetylase related to the yeast transcriptional regulator Rpd3p. Tanpakushitsu Kakusan Koso.

[39] Ahmad M, Hamid A, Hussain A, Majeed R, Qurishi Y, Bhat JA, Najar RA, Qazi AK, Zargar MA, Singh SK, Saxena AK (2012). Understanding histone deacetylases in the cancer development and treatment: an epigenetic perspective of cancer chemotherapy. DNA Cell Biol.

[40] Haberland M, Montgomery RL, Olson EN (2009). The many roles of histone deacetylases in development and physiology: implications for disease and therapy. Nat Rev Genet.

[41] Gray SG, Ekstrom TJ (2001). The human histone deacetylase family. Exp Cell Res.

[42] Gao L, Cueto MA, Asselbergs F, Atadja P (2002). Cloning and functional characterization of HDAC11, a novel member of the human histone deacetylase family. J Biol Chem.

[43] Bolden JE, Peart MJ, Johnstone RW (2006). Anticancer activities of histone deacetylase inhibitors. Nat Rev Drug Discov.

[44] New M, Olzscha H, La Thangue NB (2012). HDAC inhibitor-based therapies: can we interpret the code?. Mol Oncol.

[45] Wolffe AP, Guschin D (2000). Review: chromatin structural features and targets that regulate transcription. J Struct Biol.

[46] Eickbush TH, Moudrianakis EN (1978). The histone core complex: an octamer assembled by two sets of protein-protein interactions. Biochemistry-us.

[47] Tessarz P, Kouzarides T (2014). Histone core modifications regulating nucleosome structure and dynamics. Nat Rev Mol Cell Biol.

[48] Lee DY, Hayes JJ, Pruss D, Wolffe AP (1993). A positive role for histone acetylation in transcription factor access to nucleosomal DNA. Cell.

[49] Fenley AT, Anandakrishnan R, Kidane YH, Onufriev AV. Modulation of nucleosomal DNA accessibility via charge-altering post-translational modifications in histone core.; 2018. p 11. 10.1186/s13072-018-0181-5 PMC5856334 29548294

[50] Arrowsmith CH, Bountra C, Fish PV, Lee K, Schapira M (2012). Epigenetic protein families: a new frontier for drug discovery. Nat Rev Drug Discov.

[51] Jain AK, Barton MC (2017). Bromodomain Histone Readers and Cancer. J Mol Biol.

[52] Agalioti T, Chen G, Thanos D (2002). Deciphering the transcriptional histone acetylation code for a human gene. Cell.

[53] Peterson CL (2002). Chromatin remodeling: nucleosomes bulging at the seams. Curr Biol.

[54] Merika M, Thanos D (2001). Enhanceosomes. Curr Opin Genet Dev.

[55] Marks P, Rifkind RA, Richon VM, Breslow R, Miller T, Kelly WK (2001). Histone deacetylases and cancer: causes and therapies. Nat Rev Cancer.

[56] Gu W, Roeder RG (1997). Activation of p53 sequence-specific DNA binding by acetylation of the p53 C-terminal domain. Cell.

[57] Martinez-Balbas MA, Bauer UM, Nielsen SJ, Brehm A, Kouzarides T (2000). Regulation of E2F1 activity by acetylation. Embo J.

[58] Patel JH, Du Y, Ard PG, Phillips C, Carella B, Chen CJ, Rakowski C, Chatterjee C, Lieberman PM, Lane WS, Blobel GA, McMahon SB (2004). The c-MYC oncoprotein is a substrate of the acetyltransferases hGCN5/PCAF and TIP60. Mol Cell Biol.

[59] Chen L, Fischle W, Verdin E, Greene WC (2001). Duration of nuclear NF-kappaB action regulated by reversible acetylation. Science.

[60] Jeong JW, Bae MK, Ahn MY, Kim SH, Sohn TK, Bae MH, Yoo MA, Song EJ, Lee KJ, Kim KW (2002). Regulation and destabilization of HIF-1alpha by ARD1-mediated acetylation. Cell.

[61] Wang C, Fu M, Angeletti RH, Siconolfi-Baez L, Reutens AT, Albanese C, Lisanti MP, Katzenellenbogen BS, Kato S, Hopp T, Fuqua SA, Lopez GN, Kushner PJ, Pestell RG (2001). Direct acetylation of the estrogen receptor alpha hinge region by p300 regulates transactivation and hormone sensitivity. J Biol Chem.

[62] Gaughan L, Logan IR, Cook S, Neal DE, Robson CN (2002). Tip60 and histone deacetylase 1 regulate androgen receptor activity through changes to the acetylation status of the receptor. J Biol Chem.

[63] Glozak MA, Sengupta N, Zhang X, Seto E (2005). Acetylation and deacetylation of non-histone proteins. Gene.

[64] Bannister AJ, Kouzarides T (2011). Regulation of chromatin by histone modifications. Cell Res.

[65] Minucci S, Pelicci PG (2006). Histone deacetylase inhibitors and the promise of epigenetic (and more) treatments for cancer. Nat Rev Cancer.

[66] Mai A, Massa S, Rotili D, Cerbara I, Valente S, Pezzi R, Simeoni S, Ragno R (2005). Histone deacetylation in epigenetics: an attractive target for anticancer therapy. MED RES REV.

[67] Kumar S, Ahmad MK, Waseem M, Pandey AK. Drug Targets for Cancer Treatment: An Overview. Med Chem. 2015;5:115–23.

[68] Bassett SA, Barnett MP (2014). The role of dietary histone deacetylases (HDACs) inhibitors in health and disease. Nutrients.

[69] Vidanes GM, Bonilla CY, Toczyski DP (2005). Complicated tails: histone modifications and the DNA damage response. Cell.

[70] Polo SE, Almouzni G (2005). Histone metabolic pathways and chromatin assembly factors as proliferation markers. Cancer Lett.

[71] Singh AK, Bishayee A, Pandey AK. Targeting Histone Deacetylases with Natural and Synthetic Agents: An Emerging Anticancer Strategy. Nutrients. 2018;10. 10.3390/nu10060731 PMC6024317 29882797

[72] Petta V, Gkiozos I, Strimpakos A, Syrigos K (2013). Histones and lung cancer: Are the histone deacetylases a promising therapeutic target?. Cancer Chemother Pharmacol.

[73] Cao C, Vasilatos SN, Bhargava R, Fine JL, Oesterreich S, Davidson NE, Huang Y (2017). Functional interaction of histone deacetylase 5 (HDAC5) and lysine-specific demethylase 1 (LSD1) promotes breast cancer progression. Oncogene.

[74] Rikimaru T, Taketomi A, Yamashita Y, Shirabe K, Hamatsu T, Shimada M, Maehara Y (2007). Clinical significance of histone deacetylase 1 expression in patients with hepatocellular carcinoma. Oncology.

[75] Insinga A, Monestiroli S, Ronzoni S, Carbone R, Pearson M, Pruneri G, Viale G, Appella E, Pelicci P, Minucci S (2004). Impairment of p53 acetylation, stability and function by an oncogenic transcription factor. Embo J.

[76] Luo J, Su F, Chen D, Shiloh A, Gu W (2000). Deacetylation of p53 modulates its effect on cell growth and apoptosis. Nature.

[77] Halkidou K, Gaughan L, Cook S, Leung HY, Neal DE, Robson CN (2004). Upregulation and nuclear recruitment of HDAC1 in hormone refractory prostate cancer. Prostate.

[78] Fischle W (2009). Tip60-ing the balance in DSB repair. Nat Cell Biol.

[79] Yi J, Huang X, Yang Y, Zhu WG, Gu W, Luo J (2014). Regulation of histone acetyltransferase TIP60 function by histone deacetylase 3. J Biol Chem.

[80] Ikura T, Ogryzko VV, Grigoriev M, Groisman R, Wang J, Horikoshi M, Scully R, Qin J, Nakatani Y (2000). Involvement of the TIP60 histone acetylase complex in DNA repair and apoptosis. Cell.

[81] Roos WP, Krumm A (2016). The multifaceted influence of histone deacetylases on DNA damage signalling and DNA repair. Nucleic Acids Res.

[82] Kovacs JJ, Murphy PJ, Gaillard S, Zhao X, Wu JT, Nicchitta CV, Yoshida M, Toft DO, Pratt WB, Yao TP (2005). HDAC6 regulates Hsp90 acetylation and chaperone-dependent activation of glucocorticoid receptor. Mol Cell.

[83] Park JH, Kim SH, Choi MC, Lee J, Oh DY, Im SA, Bang YJ, Kim TY (2008). Class II histone deacetylases play pivotal roles in heat shock protein 90-mediated proteasomal degradation of vascular endothelial growth factor receptors. Biochem Biophys Res Commun.

[84] Mottet D, Bellahcene A, Pirotte S, Waltregny D, Deroanne C, Lamour V, Lidereau R, Castronovo V (2007). Histone deacetylase 7 silencing alters endothelial cell migration, a key step in angiogenesis. Circ Res.

[85] Shakespear MR, Halili MA, Irvine KM, Fairlie DP, Sweet MJ (2011). Histone deacetylases as regulators of inflammation and immunity. Trends Immunol.

[86] Deng WG, Zhu Y, Wu KK (2004). Role of p300 and PCAF in regulating cyclooxygenase-2 promoter activation by inflammatory mediators. Blood.

[87] Choi YS, Jeong S (2005). PI3-kinase and PDK-1 regulate HDAC1-mediated transcriptional repression of transcription factor NF-kappaB. Mol Cells.

[88] Nusinzon I, Horvath CM (2006). Positive and negative regulation of the innate antiviral response and beta interferon gene expression by deacetylation. Mol Cell Biol.

[89] Duan H, Heckman CA, Boxer LM (2005). Histone deacetylase inhibitors down-regulate bcl-2 expression and induce apoptosis in t(14;18) lymphomas. Mol Cell Biol.

[90] Weichert W, Roske A, Niesporek S, Noske A, Buckendahl AC, Dietel M, Gekeler V, Boehm M, Beckers T, Denkert C (2008). Class I histone deacetylase expression has independent prognostic impact in human colorectal cancer: specific role of class I histone deacetylases in vitro and in vivo. Clin Cancer Res.

[91] Osada H, Tatematsu Y, Saito H, Yatabe Y, Mitsudomi T, Takahashi T (2004). Reduced expression of class II histone deacetylase genes is associated with poor prognosis in lung cancer patients. Int J Cancer.

[92] Johnstone RW (2002). Histone-deacetylase inhibitors: novel drugs for the treatment of cancer. Nat Rev Drug Discov.

[93] Zhang XD, Gillespie SK, Borrow JM, Hersey P (2004). The histone deacetylase inhibitor suberic bishydroxamate regulates the expression of multiple apoptotic mediators and induces mitochondria-dependent apoptosis of melanoma cells. Mol Cancer Ther.

[94] Chiao MT, Cheng WY, Yang YC, Shen CC, Ko JL (2013). Suberoylanilide hydroxamic acid (SAHA) causes tumor growth slowdown and triggers autophagy in glioblastoma stem cells. Autophagy.

[95] Hesham HM, Lasheen DS, Abouzid K (2018). Chimeric HDAC inhibitors: Comprehensive review on the HDAC-based strategies developed to combat cancer. Med Res Rev.

[96] Wang Z, Chen Z, Jiang G, Wu Y, Liu T, Yi Y, Zeng J, Du J, Wang H (2016). Histone deacetylase inhibitors suppress mutant p53 transcription via HDAC8/YY1 signals in triple negative breast cancer cells. Cell Signal.

[97] Gilardini Montani MS, Granato M, Santoni C, Del Porto P, Merendino N, D Orazi G, Faggioni A, Cirone M. Histone deacetylase inhibitors VPA and TSA induce apoptosis and autophagy in pancreatic cancer cells. Cell Oncol 2017;40:167-180. 10.1007/s13402-017-0314-z PMC13001582 28160167

[98] Symanowski J, Vogelzang N, Zawel L, Atadja P, Pass H, Sharma S (2009). A Histone Deacetylase Inhibitor LBH589 Downregulates XIAP in Mesothelioma Cell Lines Which is Likely Responsible for Increased Apoptosis With TRAIL. J Thorac Oncol.

[99] Rosato RR, Maggio SC, Almenara JA, Payne SG, Atadja P, Spiegel S, Dent P, Grant S (2006). RETRACTION: The Histone Deacetylase Inhibitor LAQ824 Induces Human Leukemia Cell Death through a Process Involving XIAP Down-Regulation, Oxidative Injury, and the Acid Sphingomyelinase-Dependent Generation of Ceramide. Mol Pharmacol.

[100] Xu W, Ngo L, Perez G, Dokmanovic M, Marks PA (2006). Intrinsic apoptotic and thioredoxin pathways in human prostate cancer cell response to histone deacetylase inhibitor. P Natl Acad Sci Usa.

[101] Lucas DM, Davis ME, Parthun MR, Mone AP, Kitada S, Cunningham KD, Flax EL, Wickham J, Reed JC, Byrd JC, Grever MR (2004). The histone deacetylase inhibitor MS-275 induces caspase-dependent apoptosis in B-cell chronic lymphocytic leukemia cells. Leukemia.

[102] Insinga A, Monestiroli S, Ronzoni S, Gelmetti V, Marchesi F, Viale A, Altucci L, Nervi C, Minucci S, Pelicci PG (2005). Inhibitors of histone deacetylases induce tumor-selective apoptosis through activation of the death receptor pathway. Nat Med.

[103] Singh TR, Shankar S, Srivastava RK (2005). HDAC inhibitors enhance the apoptosis-inducing potential of TRAIL in breast carcinoma. Oncogene.

[104] Fotheringham S, Epping MT, Stimson L, Khan O, Wood V, Pezzella F, Bernards R, La Thangue NB (2009). Genome-wide Loss-of-Function Screen Reveals an Important Role for the Proteasome in HDAC Inhibitor-Induced Apoptosis. Cancer Cell.

[105] Scott GK, Marden C, Xu F, Kirk L, Benz CC (2002). Transcriptional Repression of ErbB2 by Histone Deacetylase Inhibitors Detected by a Genomically Integrated ErbB2 Promoter-reporting Cell Screen. Mol Cancer Ther.

[106] Fuino L, Bali P, Wittmann S, Donapaty S, Guo F, Yamaguchi H, Wang H, Atadja P, Bhalla K (2003). Histone deacetylase inhibitor LAQ824 down-regulates Her-2 and sensitizes human breast cancer cells to trastuzumab, taxotere, gemcitabine, and epothilone B. Mol Cancer Ther.

[107] Shulak L, Beljanski V, Chiang C, Dutta SM, Van Grevenynghe J, Belgnaoui SM, Nguyên TL, Di Lenardo T, Semmes OJ, Lin R, Hiscott J (2014). Histone deacetylase inhibitors potentiate vesicular stomatitis virus oncolysis in prostate cancer cells by modulating NF-κB-dependent autophagy. J Virol.

[108] Nimmanapalli R, Fuino L, Bali P, Gasparetto M, Glozak M, Tao J, Moscinski L, Smith C, Wu J, Jove R, Atadja P, Bhalla K (2003). Histone Deacetylase Inhibitor LAQ824 Both Lowers Expression and Promotes Proteasomal Degradation of Bcr-Abl and Induces Apoptosis of Imatinib Mesylate-sensitive or -refractory Chronic Myelogenous Leukemia-Blast Crisis Cells. Cancer Res.

[109] Richon VM, Sandhoff TW, Rifkind RA, Marks PA (2000). Histone deacetylase inhibitor selectively induces p21WAF1 expression and gene-associated histone acetylation. P Natl Acad Sci Usa.

[110] Archer SY, Meng S, Shei A, Hodin RA (1998). p21(WAF1) is required for butyrate-mediated growth inhibition of human colon cancer cells. P Natl Acad Sci Usa.

[111] Xu WS, Parmigiani RB, Marks PA (2007). Histone deacetylase inhibitors: molecular mechanisms of action. Oncogene.

[112] Chen Z, Clark S, Birkeland M, Sung C, Lago A, Liu R, Kirkpatrick R, Johanson K, Winkler JD, Hu E (2002). Induction and superinduction of growth arrest and DNA damage gene 45 (GADD45) α and β messenger RNAs by histone deacetylase inhibitors trichostatin A (TSA) and butyrate in SW620 human colon carcinoma cells. Cancer Lett.

[113] Jaboin J, Wild J, Hamidi H, Khanna C, Kim CJ, Robey R, Bates SE, Thiele CJ (2002). MS-27-275, an inhibitor of histone deacetylase, has marked in vitro and in vivo antitumor activity against pediatric solid tumors. Cancer Res.

[114] Mie Lee Y, Kim S, Kim H, Jin Son M, Nakajima H, Jeong Kwon H, Kim K (2003). Inhibition of hypoxia-induced angiogenesis by FK228, a specific histone deacetylase inhibitor, via suppression of HIF-1α activity. Biochem Bioph Res Co.

[115] Kim MS, Kwon HJ, Lee YM, Baek JH, Jang J, Lee S, Moon E, Kim H, Lee S, Chung HY, Kim CW, Kim K (2001). Histone deacetylases induce angiogenesis by negative regulation of tumor suppressor genes. Nat Med.

[116] Munshi A, Kurland JF, Nishikawa T, Tanaka T, Hobbs ML, Tucker SL, Ismail S, Stevens C, Meyn RE (2005). Histone Deacetylase Inhibitors Radiosensitize Human Melanoma Cells by Suppressing DNA Repair Activity. Clin Cancer Res.

[117] Zhang Y, Carr T, Dimtchev A, Zaer N, Dritschilo A, Jung M. Attenuated DNA Damage Repair by Trichostatin A through BRCA1 Suppression. Radiat Res 2007;168:115-124, 10. 10.1667/RR0811.1 17722998

[118] Woods DM, Sodré AL, Villagra A, Sarnaik A, Sotomayor EM, Weber J (2015). HDAC Inhibition Upregulates PD-1 Ligands in Melanoma and Augments Immunotherapy with PD-1 Blockade. Cancer Immunol Res.

[119] Shen L, Ciesielski M, Ramakrishnan S, Miles KM, Ellis L, Sotomayor P, Shrikant P, Fenstermaker R, Pili R (2012). Class I histone deacetylase inhibitor entinostat suppresses regulatory T cells and enhances immunotherapies in renal and prostate cancer models. Plos One.

[120] Bojang PJ, Ramos KS (2014). The promise and failures of epigenetic therapies for cancer treatment. Cancer Treat Rev.

[121] Marks PA, Breslow R. Dimethyl sulfoxide to vorinostat: development of this histone deacetylase inhibitor as an anticancer drug.; 2007. pp 84-90. 10.1038/nbt1272 17211407

[122] Hervouet E (2018). The Promising Role of New Generation HDACis in Anti-Cancer Therapies. Ebiomedicine.

[123] Zhang T, Li J, Ma X, Yang Y, Sun W, Jin W, Wang L, He Y, Yang F, Yi Z, Hua Y, Liu M, Chen Y, Cai Z. Inhibition of HDACs-EphA2 Signaling Axis with WW437 Demonstrates Promising Preclinical Antitumor Activity in Breast Cancer.; 2018. pp 276-286. 10.1016/j.ebiom.2018.05.003 PMC6013969 29759486

[124] Douillard JY, Bennouna J, Vavasseur F, Deporte-Fety R, Thomare P, Giacalone F, Meflah K. Phase I trial of interleukin-2 and high-dose arginine butyrate in metastatic colorectal cancer.; 2000. pp 56-61. 10.1007/s002620050026 PMC11036988 10782866

[125] Su JM, Li X, Thompson P, Ou C, Ingle AM, Russell H, Lau CC, Adamson PC, Blaney SM. Phase 1 study of valproic acid in pediatric patients with refractory solid or CNS tumors: a children's oncology group report.; 2011. pp 589-597. 10.1158/1078-0432.CCR-10-0738 PMC3064523 21115653

[126] Batlevi CL, Kasamon Y, Bociek RG, Lee P, Gore L, Copeland A, Sorensen R, Ordentlich P, Cruickshank S, Kunkel L, Buglio D, Hernandez-Ilizaliturri F, Younes A. ENGAGE- 501: phase II study of entinostat (SNDX-275) in relapsed and refractory Hodgkin lymphoma.; 2016. pp 968-975. 10.3324/haematol.2016.142406 PMC4967576 27151994

[127] Younes A, Oki Y, Bociek RG, Kuruvilla J, Fanale M, Neelapu S, Copeland A, Buglio D, Galal A, Besterman J, Li Z, Drouin M, Patterson T, Ward MR, Paulus JK, Ji Y, Medeiros LJ, Martell RE. Mocetinostat for relapsed classical Hodgkin's lymphoma: an open-label, single-arm, phase 2 trial.; 2011. pp 1222-1228. 10.1016/S1470-2045(11)70265-0 PMC5042214 22033282

[128] Whittaker SJ, Demierre M, Kim EJ, Rook AH, Lerner A, Duvic M, Scarisbrick J, Reddy S, Robak T, Becker JC, Samtsov A, McCulloch W, Kim YH. Final results from a multicenter, international, pivotal study of romidepsin in refractory cutaneous T-cell lymphoma.; 2010. pp 4485-4491. 10.1200/JCO.2010.28.9066 20697094

[129] Kijima M, Yoshida M, Sugita K, Horinouchi S, Beppu T (1993). Trapoxin, an antitumor cyclic tetrapeptide, is an irreversible inhibitor of mammalian histone deacetylase. J Biol Chem.

[130] Wada CK, Frey RR, Ji Z, Curtin ML, Garland RB, Holms JH, Li J, Pease LJ, Guo J, Glaser KB, Marcotte PA, Richardson PL, Murphy SS, Bouska JJ, Tapang P, Magoc TJ, Albert DH, Davidsen SK, Michaelides MR. Alpha-keto amides as inhibitors of histone deacetylase.; 2003. pp 3331-3335. 10.1016/s0960-894x(03)00685-1 12951120

[131] Vasudevan A, Ji Z, Frey RR, Wada CK, Steinman D, Heyman HR, Guo Y, Curtin ML, Guo J, Li J, Pease L, Glaser KB, Marcotte PA, Bouska JJ, Davidsen SK, Michaelides MR. Heterocyclic ketones as inhibitors of histone deacetylase.; 2003. pp 3909-3913. 10.1016/j.bmcl.2003.09.007 14592473

[132] Das A, Henderson F, Lowe S, Wallace GC, Vandergrift WA, Lindhorst SM, Varma AK, Infinger LK, Giglio P, Banik NL, Patel SJ, Cachia D. Single agent efficacy of the HDAC inhibitor DATS in preclinical models of glioblastoma.; 2018. pp 945-952. 10.1007/s00280-018-3684-7 30209569

[133] Eckschlager T, Plch J, Stiborova M, Hrabeta J. Histone Deacetylase Inhibitors as Anticancer Drugs.; 2017. 10.3390/ijms18071414 PMC5535906 28671573

[134] Burgess AJ, Pavey S, Warrener R, Hunter LJ, Piva TJ, Musgrove EA, Saunders N, Parsons PG, Gabrielli BG (2001). Up-regulation of p21(WAF1/CIP1) by histone deacetylase inhibitors reduces their cytotoxicity. Mol Pharmacol.

[135] Coffey DC, Kutko MC, Glick RD, Butler LM, Heller G, Rifkind RA, Marks PA, Richon VM, La Quaglia MP (2001). The histone deacetylase inhibitor, CBHA, inhibits growth of human neuroblastoma xenografts in vivo, alone and synergistically with all-trans retinoic acid. Cancer Res.

[136] Yang L, Liang Q, Shen K, Ma L, An N, Deng W, Fei Z, Liu J. A novel class I histone deacetylase inhibitor, I-7ab, induces apoptosis and arrests cell cycle progression in human colorectal cancer cells.; 2015. pp 70-78. 10.1016/j.biopha.2015.02.019 25960218

[137] Wells CE, Bhaskara S, Stengel KR, Zhao Y, Sirbu B, Chagot B, Cortez D, Khabele D, Chazin WJ, Cooper A, Jacques V, Rusche J, Eischen CM, McGirt LY, Hiebert SW (2013). Inhibition of histone deacetylase 3 causes replication stress in cutaneous T cell lymphoma. Plos One.

[138] Balasubramanian S, Ramos J, Luo W, Sirisawad M, Verner E, Buggy JJ. A novel histone deacetylase 8 (HDAC8)-specific inhibitor PCI-34051 induces apoptosis in T-cell lymphomas.; 2008. pp 1026-1034. 10.1038/leu.2008.9 18256683

[139] Suzuki T, Ota Y, Ri M, Bando M, Gotoh A, Itoh Y, Tsumoto H, Tatum PR, Mizukami T, Nakagawa H, Iida S, Ueda R, Shirahige K, Miyata N. Rapid discovery of highly potent and selective inhibitors of histone deacetylase 8 using click chemistry to generate candidate libraries.; 2012. pp 9562-9575. 10.1021/jm300837y 23116147

[140] Aldana-Masangkay GI, Rodriguez-Gonzalez A, Lin T, Ikeda AK, Hsieh Y, Kim Y, Lomenick B, Okemoto K, Landaw EM, Wang D, Mazitschek R, Bradner JE, Sakamoto KM. Tubacin suppresses proliferation and induces apoptosis of acute lymphoblastic leukemia cells.; 2011. pp 1544-1555. 10.3109/10428194.2011.570821 PMC4113006 21699378

[141] Solomon JM, Pasupuleti R, Xu L, McDonagh T, Curtis R, DiStefano PS, Huber LJ (2006). Inhibition of SIRT1 catalytic activity increases p53 acetylation but does not alter cell survival following DNA damage. Mol Cell Biol.

[142] Medda F, Russell RJM, Higgins M, McCarthy AR, Campbell J, Slawin AMZ, Lane DP, Lain S, Westwood NJ (2009). Novel cambinol analogs as sirtuin inhibitors: synthesis, biological evaluation, and rationalization of activity. J Med Chem.

[143] Kalle AM, Mallika A, Badiger J, Alinakhi, Talukdar P, Sachchidanand. Inhibition of SIRT1 by a small molecule induces apoptosis in breast cancer cells.; 2010. pp 13-19. 10.1016/j.bbrc.2010.08.118 20813094

[144] Garcia PL, Miller AL, Gamblin TL, Council LN, Christein JD, Arnoletti JP, Heslin MJ, Reddy S, Richardson JH, Cui X, van Waardenburg RCAM, Bradner JE, Yang ES, Yoon KJ. JQ1 Induces DNA Damage and Apoptosis, and Inhibits Tumor Growth in a Patient-Derived Xenograft Model of Cholangiocarcinoma.; 2018. pp 107-118. 10.1158/1535-7163.MCT-16-0922 PMC5752593 29142067

[145] Zhang D, Leal AS, Carapellucci S, Zydeck K, Sporn MB, Liby KT. Chemoprevention of Preclinical Breast and Lung Cancer with the Bromodomain Inhibitor I-BET 762.; 2018. pp 143-156. 10.1158/1940-6207.CAPR-17-0264 PMC10265586 29246957

[146] Chen D, Lu T, Yan Z, Lu W, Zhou F, Lyu X, Xu B, Jiang H, Chen K, Luo C, Zhao Y. Discovery, structural insight, and bioactivities of BY27 as a selective inhibitor of the second bromodomains of BET proteins.; 2019. p 111633. 10.1016/j.ejmech.2019.111633 31461688

[147] Bertrand P (2010). Inside HDAC with HDAC inhibitors. Eur J Med Chem.

[148] Sarfstein R, Bruchim I, Fishman A, Werner H (2011). The mechanism of action of the histone deacetylase inhibitor vorinostat involves interaction with the insulin-like growth factor signaling pathway. Plos One.

[149] Ma T, Galimberti F, Erkmen CP, Memoli V, Chinyengetere F, Sempere L, Beumer JH, Anyang BN, Nugent W, Johnstone D, Tsongalis GJ, Kurie JM, Li H, Direnzo J, Guo Y, Freemantle SJ, Dragnev KH, Dmitrovsky E (2013). Comparing histone deacetylase inhibitor responses in genetically engineered mouse lung cancer models and a window of opportunity trial in patients with lung cancer. Mol Cancer Ther.

[150] Doi T, Hamaguchi T, Shirao K, Chin K, Hatake K, Noguchi K, Otsuki T, Mehta A, Ohtsu A (2013). Evaluation of safety, pharmacokinetics, and efficacy of vorinostat, a histone deacetylase inhibitor, in the treatment of gastrointestinal (GI) cancer in a phase I clinical trial. Int J Clin Oncol.

[151] Yin D, Ong JM, Hu J, Desmond JC, Kawamata N, Konda BM, Black KL, Koeffler HP (2007). Suberoylanilide hydroxamic acid, a histone deacetylase inhibitor: effects on gene expression and growth of glioma cells in vitro and in vivo. Clin Cancer Res.

[152] Bezecny P (2014). Histone deacetylase inhibitors in glioblastoma: pre-clinical and clinical experience. Med Oncol.

[153] Soragni E, Xu C, Plasterer HL, Jacques V, Rusche JR, Gottesfeld JM (2012). Rationale for the development of 2-aminobenzamide histone deacetylase inhibitors as therapeutics for Friedreich ataxia. J Child Neurol.

[154] Minami J, Suzuki R, Mazitschek R, Gorgun G, Ghosh B, Cirstea D, Hu Y, Mimura N, Ohguchi H, Cottini F, Jakubikova J, Munshi NC, Haggarty SJ, Richardson PG, Hideshima T, Anderson KC (2014). Histone deacetylase 3 as a novel therapeutic target in multiple myeloma. Leukemia.

[155] Yang M, Dang X, Tan Y, Wang M, Li X, Li G (2018). I-7ab inhibited the growth of TNBC cells via targeting HDAC3 and promoting the acetylation of p53. Biomed Pharmacother.

[156] Verdin E, Ott M. 50 years of protein acetylation: from gene regulation to epigenetics, metabolism and beyond.; 2015. pp 258-264. 10.1038/nrm3931 25549891

[157] Filippakopoulos P, Qi J, Picaud S, Shen Y, Smith WB, Fedorov O, Morse EM, Keates T, Hickman TT, Felletar I, Philpott M, Munro S, McKeown MR, Wang Y, Christie AL, West N, Cameron MJ, Schwartz B, Heightman TD, La Thangue N, French CA, Wiest O, Kung AL, Knapp S, Bradner JE. Selective inhibition of BET bromodomains.; 2010. pp 1067-1073. 10.1038/nature09504 PMC3010259 20871596

[158] French CA. Small-Molecule Targeting of BET Proteins in Cancer.; 2016. pp 21-58. 10.1016/bs.acr.2016.04.001 27451123

[159] Cai X, Zhai H, Wang J, Forrester J, Qu H, Yin L, Lai C, Bao R, Qian C. Discovery of 7-(4-(3-ethynylphenylamino)-7-methoxyquinazolin-6-yloxy)-N-hydroxyheptanamide (CUDc-101) as a potent multi-acting HDAC, EGFR, and HER2 inhibitor for the treatment of cancer.; 2010. pp 2000-2009. 10.1021/jm901453q 20143778

[160] Wang J, Pursell NW, Samson MES, Atoyan R, Ma AW, Selmi A, Xu W, Cai X, Voi M, Savagner P, Lai C. Potential advantages of CUDC-101, a multitargeted HDAC, EGFR, and HER2 inhibitor, in treating drug resistance and preventing cancer cell migration and invasion.; 2013. pp 925-936. 10.1158/1535-7163.MCT-12-1045 23536719

[161] Ellis HP, Greenslade M, Powell B, Spiteri I, Sottoriva A, Kurian KM. Current Challenges in Glioblastoma: Intratumour Heterogeneity, Residual Disease, and Models to Predict Disease Recurrence.; 2015. p 251. 10.3389/fonc.2015.00251 PMC4644939 26636033

[162] Zorzan M, Giordan E, Redaelli M, Caretta A, Mucignat-Caretta C. Molecular targets in glioblastoma.; 2015. pp 1407-1420. 10.2217/fon.15.22 25952786

[163] Yang R, Wu Y, Wang M, Sun Z, Zou J, Zhang Y, Cui H. HDAC9 promotes glioblastoma growth via TAZ-mediated EGFR pathway activation.; 2015. pp 7644-7656. 10.18632/oncotarget.3223 PMC4480706 25760078

[164] Pastori C, Daniel M, Penas C, Volmar C, Johnstone AL, Brothers SP, Graham RM, Allen B, Sarkaria JN, Komotar RJ, Wahlestedt C, Ayad NG. BET bromodomain proteins are required for glioblastoma cell proliferation.; 2014. pp 611-620. 10.4161/epi.27906 PMC4121371 24496381

[165] Bajbouj K, Mawrin C, Hartig R, Schulze-Luehrmann J, Wilisch-Neumann A, Roessner A, Schneider-Stock R. P53-dependent antiproliferative and pro-apoptotic effects of trichostatin A (TSA) in glioblastoma cells.; 2012. pp 503-516. 10.1007/s11060-011-0791-2 22270849

[166] Sawa H, Murakami H, Ohshima Y, Sugino T, Nakajyo T, Kisanuki T, Tamura Y, Satone A, Ide W, Hashimoto I, Kamada H. Histone deacetylase inhibitors such as sodium butyrate and trichostatin A induce apoptosis through an increase of the bcl-2-related protein Bad.; 2001. pp 109-114. 10.1007/BF02479423 11908866

[167] Kusaczuk M, Krętowski R, Bartoszewicz M, Cechowska-Pasko M. Phenylbutyrate-a pan-HDAC inhibitor-suppresses proliferation of glioblastoma LN-229 cell line.; 2016. pp 931-942. 10.1007/s13277-015-3781-8 PMC4841856 26260271

[168] Sawa H, Murakami H, Kumagai M, Nakasato M, Yamauchi S, Matsuyama N, Tamura Y, Satone A, Ide W, Hashimoto I, Kamada H. Histone deacetylase inhibitor, FK228, induces apoptosis and suppresses cell proliferation of human glioblastoma cells in vitro and in vivo.; 2004. pp 523-531. 10.1007/s00401-004-0841-3 15024582

[169] Wallace GC, Haar CP, Vandergrift WA, Giglio P, Dixon-Mah YN, Varma AK, Ray SK, Patel SJ, Banik NL, Das A. Multi-targeted DATS prevents tumor progression and promotes apoptosis in ectopic glioblastoma xenografts in SCID mice via HDAC inhibition.; 2013. pp 43-50. 10.1007/s11060-013-1165-8 PMC4312109 23754639

[170] Kusaczuk M, Krętowski R, Stypułkowska A, Cechowska-Pasko M. Molecular and cellular effects of a novel hydroxamate-based HDAC inhibitor - belinostat - in glioblastoma cell lines: a preliminary report.; 2016. pp 552-564. 10.1007/s10637-016-0372-5 PMC5007275 27468826

[171] Huang W, Lin C, Lee C, Chi L, Chao Y, Wang H, Chiou B, Chen T, Huang C, Chen C. NBM-HD-3, a novel histone deacetylase inhibitor with anticancer activity through modulation of PTEN and AKT in brain cancer cells.; 2011. pp 156-167. 10.1016/j.jep.2011.04.034 21530633

[172] Sharma V, Koul N, Joseph C, Dixit D, Ghosh S, Sen E. HDAC inhibitor, scriptaid, induces glioma cell apoptosis through JNK activation and inhibits telomerase activity.; 2010. pp 2151-2161. 10.1111/j.1582-4934.2009.00844.x PMC3823006 19583803

[173] Eyüpoglu IY, Hahnen E, Tränkle C, Savaskan NE, Siebzehnrübl FA, Buslei R, Lemke D, Wick W, Fahlbusch R, Blümcke I. Experimental therapy of malignant gliomas using the inhibitor of histone deacetylase MS-275.; 2006. pp 1248-1255. 10.1158/1535-7163.MCT-05-0533 16731757

[174] Papi A, Ferreri AM, Rocchi P, Guerra F, Orlandi M (2010). Epigenetic modifiers as anticancer drugs: effectiveness of valproic acid in neural crest-derived tumor cells. Anticancer Res.

[175] Jin H, Liang L, Liu L, Deng W, Liu J. HDAC inhibitor DWP0016 activates p53 transcription and acetylation to inhibit cell growth in U251 glioblastoma cells.; 2013. pp 1498-1509. 10.1002/jcb.24491 23297003

[176] Pastorino O, Gentile MT, Mancini A, Del Gaudio N, Di Costanzo A, Bajetto A, Franco P, Altucci L, Florio T, Stoppelli MP, Colucci-D'Amato L (2019). Histone Deacetylase Inhibitors Impair Vasculogenic Mimicry from Glioblastoma Cells. Cancers.

[177] Sawa H, Murakami H, Ohshima Y, Murakami M, Yamazaki I, Tamura Y, Mima T, Satone A, Ide W, Hashimoto I, Kamada H. Histone deacetylase inhibitors such as sodium butyrate and trichostatin A inhibit vascular endothelial growth factor (VEGF) secretion from human glioblastoma cells.; 2002. pp 77-81. 10.1007/BF02478931 12622137

[178] Yao Z, Li W, Hua F, Cheng H, Zhao M, Sun X, Qin Y, Li J (2017). LBH589 Inhibits Glioblastoma Growth and Angiogenesis Through Suppression of HIF-1α Expression. J Neuropathol Exp Neurol.

[179] Orzan F, Pellegatta S, Poliani PL, Pisati F, Caldera V, Menghi F, Kapetis D, Marras C, Schiffer D, Finocchiaro G. Enhancer of Zeste 2 (EZH2) is up-regulated in malignant gliomas and in glioma stem-like cells.; 2011. pp 381-394. 10.1111/j.1365-2990.2010.01132.x 20946108

[180] Nam JH, Cho H, Kang H, Lee J, Jung M, Chang Y, Kim K, Hoe H. A Mercaptoacetamide-Based Class II Histone Deacetylase Inhibitor Suppresses Cell Migration and Invasion in Monomorphic Malignant Human Glioma Cells by Inhibiting FAK/STAT3 Signaling.; 2017. pp 4672-4685. 10.1002/jcb.26133 28498494

[181] Chen C, Weng S, Tseng P, Lin H, Chen C. Histone acetylation-independent effect of histone deacetylase inhibitors on Akt through the reshuffling of protein phosphatase 1 complexes.; 2005. pp 38879-38887. 10.1074/jbc.M505733200 16186112

[182] Hsu C, Chang W, Hsu T, Liu J, Yeh S, Wang J, Liou J, Ko C, Chang K, Chuang J. Suberoylanilide hydroxamic acid represses glioma stem-like cells.; 2016. p 81. 10.1186/s12929-016-0296-6 PMC5116136 27863490

[183] Pastori C, Kapranov P, Penas C, Peschansky V, Volmar C, Sarkaria JN, Bregy A, Komotar R, St Laurent G, Ayad NG, Wahlestedt C. The Bromodomain protein BRD4 controls HOTAIR, a long noncoding RNA essential for glioblastoma proliferation.; 2015. pp 8326-8331. 10.1073/pnas.1424220112 PMC4500283 26111795

[184] Alvarez AA, Field M, Bushnev S, Longo MS, Sugaya K. The effects of histone deacetylase inhibitors on glioblastoma-derived stem cells.; 2015. 10.1007/s12031-014-0329-0 24874578

[185] Galanis E, Jaeckle KA, Maurer MJ, Reid JM, Ames MM, Hardwick JS, Reilly JF, Loboda A, Nebozhyn M, Fantin VR, Richon VM, Scheithauer B, Giannini C, Flynn PJ, Moore DF, Zwiebel J, Buckner JC. Phase II trial of vorinostat in recurrent glioblastoma multiforme: a north central cancer treatment group study.; 2009. pp 2052-2058. 10.1200/JCO.2008.19.0694 PMC2669764 19307505

[186] Iwamoto FM, Lamborn KR, Kuhn JG, Wen PY, Yung WKA, Gilbert MR, Chang SM, Lieberman FS, Prados MD, Fine HA. A phase I/II trial of the histone deacetylase inhibitor romidepsin for adults with recurrent malignant glioma: North American Brain Tumor Consortium Study 03-03.; 2011. pp 509-516. 10.1093/neuonc/nor017 PMC3093337 21377994

[187] Wu Y, Dong L, Bao S, Wang M, Yun Y, Zhu R. FK228 augmented temozolomide sensitivity in human glioma cells by blocking PI3K/AKT/mTOR signal pathways.; 2016. pp 462-469. 10.1016/j.biopha.2016.09.051 27685789

[188] Bangert A, Häcker S, Cristofanon S, Debatin K, Fulda S. Chemosensitization of glioblastoma cells by the histone deacetylase inhibitor MS275.; 2011. pp 494-499. 10.1097/CAD.0b013e32834631e0 21566522

[189] Staberg M, Michaelsen SR, Rasmussen RD, Villingshøj M, Poulsen HS, Hamerlik P. Inhibition of histone deacetylases sensitizes glioblastoma cells to lomustine.; 2017. pp 21-32. 10.1007/s13402-016-0301-9 PMC13001581 27766591

[190] Zhang Z, Wang Y, Chen J, Tan Q, Xie C, Li C, Zhan W, Wang M. Silencing of histone deacetylase 2 suppresses malignancy for proliferation, migration, and invasion of glioblastoma cells and enhances temozolomide sensitivity.; 2016. pp 1289-1296. 10.1007/s00280-016-3188-2 27832326

[191] Li Z, Li Q, Chen L, Chen B, Wang B, Zhang X, Li W. Histone Deacetylase Inhibitor RGFP109 Overcomes Temozolomide Resistance by Blocking NF-κB-Dependent Transcription in Glioblastoma Cell Lines.; 2016. pp 3192-3205. 10.1007/s11064-016-2043-5 27632183

[192] Urdiciain A, Erausquin E, Meléndez B, Rey JA, Idoate MA, Castresana JS. Tubastatin A, an inhibitor of HDAC6, enhances temozolomide-induced apoptosis and reverses the malignant phenotype of glioblastoma cells.; 2019. pp 1797-1808. 10.3892/ijo.2019.4739 30864703

[193] Li Z, Zhang C, Zhang Y, Chen L, Chen B, Li Q, Zhang X, Li W. A novel HDAC6 inhibitor Tubastatin A: Controls HDAC6-p97/VCP-mediated ubiquitination-autophagy turnover and reverses Temozolomide-induced ER stress-tolerance in GBM cells.; 2017. pp 89-99. 10.1016/j.canlet.2017.01.025 28131906

[194] de Andrade PV, Andrade AF, de Paula Queiroz RG, Scrideli CA, Tone LG, Valera ET. The histone deacetylase inhibitor PCI-24781 as a putative radiosensitizer in pediatric glioblastoma cell lines.; 2016. p 31. 10.1186/s12935-016-0306-5 PMC4835828 27095947

[195] Festuccia C, Mancini A, Colapietro A, Gravina GL, Vitale F, Marampon F, Delle Monache S, Pompili S, Cristiano L, Vetuschi A, Tombolini V, Chen Y, Mehrling T. The first-in-class alkylating deacetylase inhibitor molecule tinostamustine shows antitumor effects and is synergistic with radiotherapy in preclinical models of glioblastoma.; 2018. p 32. 10.1186/s13045-018-0576-6 PMC5830080 29486795

[196] Kim JH, Shin JH, Kim IH. Susceptibility and radiosensitization of human glioblastoma cells to trichostatin A, a histone deacetylase inhibitor.; 2004. pp 1174-1180. 10.1016/j.ijrobp.2004.03.001 15234053

[197] Liu J, Yu C, Hung P, Hsin L, Chern J. High-selective HDAC6 inhibitor promotes HDAC6 degradation following autophagy modulation and enhanced antitumor immunity in glioblastoma.; 2019. pp 458-471. 10.1016/j.bcp.2019.03.023 30885763

[198] Singh MM, Manton CA, Bhat KP, Tsai W, Aldape K, Barton MC, Chandra J. Inhibition of LSD1 sensitizes glioblastoma cells to histone deacetylase inhibitors.; 2011. pp 894-903. 10.1093/neuonc/nor049 PMC3145466 21653597

[199] De La Rosa J, Urdiciain A, Zazpe I, Zelaya MV, Meléndez B, Rey JA, Idoate MA, Castresana JS. The synergistic effect of DZ-NEP, panobinostat and temozolomide reduces clonogenicity and induces apoptosis in glioblastoma cells.; 2020. pp 283-300. 10.3892/ijo.2019.4905 31746375

[200] Meng W, Wang B, Mao W, Wang J, Zhao Y, Li Q, Zhang C, Tang Y, Ma J. Enhanced efficacy of histone deacetylase inhibitor combined with bromodomain inhibitor in glioblastoma.; 2018. p 241. 10.1186/s13046-018-0916-y PMC6167847 30285808

[201] Zhang Y, Ishida CT, Ishida W, Lo SL, Zhao J, Shu C, Bianchetti E, Kleiner G, Sanchez-Quintero MJ, Quinzii CM, Westhoff M, Karpel-Massler G, Canoll P, Siegelin MD. Combined HDAC and Bromodomain Protein Inhibition Reprograms Tumor Cell Metabolism and Elicits Synthetic Lethality in Glioblastoma.; 2018. pp 3941-3954. 10.1158/1078-0432.CCR-18-0260 PMC6095717 29764852

[202] Marino A, Sofiadis A, Baryawno N, Johnsen JI, Larsson C, Vukojević V, Ekström TJ. Enhanced effects by 4-phenylbutyrate in combination with RTK inhibitors on proliferation in brain tumor cell models.; 2011. pp 208-212. 10.1016/j.bbrc.2011.06.141 21726539

[203] Liffers K, Kolbe K, Westphal M, Lamszus K, Schulte A. Histone Deacetylase Inhibitors Resensitize EGFR/EGFRvIII-Overexpressing, Erlotinib-Resistant Glioblastoma Cells to Tyrosine Kinase Inhibition.; 2016. pp 29-40. 10.1007/s11523-015-0372-y 26032687

[204] Sarcar B, Kahali S, Chinnaiyan P. Vorinostat enhances the cytotoxic effects of the topoisomerase I inhibitor SN38 in glioblastoma cell lines.; 2010. pp 201-207. 10.1007/s11060-010-0127-7 20135194

[205] Bieler A, Mantwill K, Dravits T, Bernshausen A, Glockzin G, Köhler-Vargas N, Lage H, Gansbacher B, Holm PS. Novel three-pronged strategy to enhance cancer cell killing in glioblastoma cell lines: histone deacetylase inhibitor, chemotherapy, and oncolytic adenovirus dl520.; 2006. pp 55-70. 10.1089/hum.2006.17.55 16409125

[206] Berghauser Pont LME, Kleijn A, Kloezeman JJ, van den Bossche W, Kaufmann JK, de Vrij J, Leenstra S, Dirven CMF, Lamfers MLM. The HDAC Inhibitors Scriptaid and LBH589 Combined with the Oncolytic Virus Delta24-RGD Exert Enhanced Anti-Tumor Efficacy in Patient-Derived Glioblastoma Cells.; 2015. p e127058. 10.1371/journal.pone.0127058 PMC4436250 25993039

[207] Chang Y, Huang L, Chen Y, Wang Y, Hueng D, Huang S. The synergistic effects of valproic acid and fluvastatin on apoptosis induction in glioblastoma multiforme cell lines.; 2017. pp 155-163. 10.1016/j.biocel.2017.10.003 29017950

[208] Taylor MA, Khathayer F, Ray SK. Quercetin and Sodium Butyrate Synergistically Increase Apoptosis in Rat C6 and Human T98G Glioblastoma Cells Through Inhibition of Autophagy.; 2019. pp 1715-1725. 10.1007/s11064-019-02802-8 31011879

[209] Zhang G, Gan Y. Synergistic antitumor effects of the combined treatment with an HDAC6 inhibitor and a COX-2 inhibitor through activation of PTEN.; 2017. pp 2657-2666. 10.3892/or.2017.5981 PMC5780018 29048666

[210] Meng W, Wang B, Mao W, Wang J, Zhao Y, Li Q, Zhang C, Ma J. Enhanced efficacy of histone deacetylase inhibitor panobinostat combined with dual PI3K/mTOR inhibitor BEZ235 against glioblastoma.; 2019. 10.18999/nagjms.81.1.93 PMC6433629 30962658

[211] Singh MM, Johnson B, Venkatarayan A, Flores ER, Zhang J, Su X, Barton M, Lang F, Chandra J. Preclinical activity of combined HDAC and KDM1A inhibition in glioblastoma.; 2015. pp 1463-1473. 10.1093/neuonc/nov041 PMC4648298 25795306

[212] Rasmussen RD, Gajjar MK, Jensen KE, Hamerlik P. Enhanced efficacy of combined HDAC and PARP targeting in glioblastoma.; 2016. pp 751-763. 10.1016/j.molonc.2015.12.014 PMC5423160 26794465

[213] Ghiaseddin A, Reardon D, Massey W, Mannerino A, Lipp ES, Herndon JE, McSherry F, Desjardins A, Randazzo D, Friedman HS, Peters KB. Phase II Study of Bevacizumab and Vorinostat for Patients with Recurrent World Health Organization Grade 4 Malignant Glioma.; 2018. pp 121-157. 10.1634/theoncologist.2017-0501 PMC5813746 29133513

[214] Peters KB, Lipp ES, Miller E, Herndon JE, McSherry F, Desjardins A, Reardon DA, Friedman HS. Phase I/II trial of vorinostat, bevacizumab, and daily temozolomide for recurrent malignant gliomas.; 2018. pp 349-356. 10.1007/s11060-017-2724-1 29264836

[215] Lee EQ, Puduvalli VK, Reid JM, Kuhn JG, Lamborn KR, Cloughesy TF, Chang SM, Drappatz J, Yung WKA, Gilbert MR, Robins HI, Lieberman FS, Lassman AB, McGovern RM, Xu J, Desideri S, Ye X, Ames MM, Espinoza-Delgado I, Prados MD, Wen PY. Phase I study of vorinostat in combination with temozolomide in patients with high-grade gliomas: North American Brain Tumor Consortium Study 04-03.; 2012. pp 6032-6039. 10.1158/1078-0432.CCR-12-1841 PMC3947570 22923449

[216] Galanis E, Anderson SK, Miller CR, Sarkaria JN, Jaeckle K, Buckner JC, Ligon KL, Ballman KV, Moore DF, Nebozhyn M, Loboda A, Schiff D, Ahluwalia MS, Lee EQ, Gerstner ER, Lesser GJ, Prados M, Grossman SA, Cerhan J, Giannini C, Wen PY. Phase I/II trial of vorinostat combined with temozolomide and radiation therapy for newly diagnosed glioblastoma: results of Alliance N0874/ABTC 02.; 2018. pp 546-556. 10.1093/neuonc/nox161 PMC5909661 29016887

[217] Friday BB, Anderson SK, Buckner J, Yu C, Giannini C, Geoffroy F, Schwerkoske J, Mazurczak M, Gross H, Pajon E, Jaeckle K, Galanis E. Phase II trial of vorinostat in combination with bortezomib in recurrent glioblastoma: a north central cancer treatment group study.; 2012. pp 215-221. 10.1093/neuonc/nor198 PMC3266383 22090453

[218] Chinnaiyan P, Chowdhary S, Potthast L, Prabhu A, Tsai Y, Sarcar B, Kahali S, Brem S, Yu HM, Rojiani A, Murtagh R, Pan E. Phase I trial of vorinostat combined with bevacizumab and CPT-11 in recurrent glioblastoma.; 2012. 10.1093/neuonc/nor187 PMC3246000 22028388

[219] Lee EQ, Reardon DA, Schiff D, Drappatz J, Muzikansky A, Grimm SA, Norden AD, Nayak L, Beroukhim R, Rinne ML, Chi AS, Batchelor TT, Hempfling K, McCluskey C, Smith KH, Gaffey SC, Wrigley B, Ligon KL, Raizer JJ, Wen PY. Phase II study of panobinostat in combination with bevacizumab for recurrent glioblastoma and anaplastic glioma.; 2015. pp 862-867. 10.1093/neuonc/nou350 PMC4483124 25572329

[220] Watanabe S, Kuwabara Y, Suehiro S, Yamashita D, Tanaka M, Tanaka A, Ohue S, Araki H. Valproic acid reduces hair loss and improves survival in patients receiving temozolomide-based radiation therapy for high-grade glioma.; 2017. pp 357-363. 10.1007/s00228-016-2167-1 27889835

[221] Barker CA, Bishop AJ, Chang M, Beal K, Chan TA. Valproic acid use during radiation therapy for glioblastoma associated with improved survival.; 2013. pp 504-509. 10.1016/j.ijrobp.2013.02.012 PMC4667941 23523186

[222] Krauze AV, Myrehaug SD, Chang MG, Holdford DJ, Smith S, Shih J, Tofilon PJ, Fine HA, Camphausen K. A Phase 2 Study of Concurrent Radiation Therapy, Temozolomide, and the Histone Deacetylase Inhibitor Valproic Acid for Patients With Glioblastoma.; 2015. pp 986-992. 10.1016/j.ijrobp.2015.04.038 PMC4510472 26194676

[223] Drummond DC, Noble CO, Kirpotin DB, Guo Z, Scott GK, Benz CC. Clinical development of histone deacetylase inhibitors as anticancer agents.; 2005. pp 495-528. 10.1146/annurev.pharmtox.45.120403.095825 15822187

[224] Thurn KT, Thomas S, Moore A, Munster PN. Rational therapeutic combinations with histone deacetylase inhibitors for the treatment of cancer.; 2011. pp 263-283. 10.2217/fon.11.2 PMC3127396 21345145

[225] Lee DH, Ryu H, Won H, Kwon SH. Advances in epigenetic glioblastoma therapy.; 2017. pp 18577-18589. 10.18632/oncotarget.14612 PMC5392350 28099914

[226] Adamopoulou E, Naumann U. HDAC inhibitors and their potential applications to glioblastoma therapy.; 2013. p e25219. 10.4161/onci.25219 PMC3805657 24167760

